# Liquid biopsies in patients with diffuse glioma

**DOI:** 10.1007/s00401-015-1399-y

**Published:** 2015-02-27

**Authors:** Myron G. Best, Nik Sol, Sebastiaan Zijl, Jaap C. Reijneveld, Pieter Wesseling, Thomas Wurdinger

**Affiliations:** 1 Neuro-oncology Research Group, VU University Medical Center, Cancer Center Amsterdam, CCA Room 3.20, Boelelaan 1117, 1081 HV Amsterdam, The Netherlands; 2 Department of Neurosurgery, VU University Medical Center, Cancer Center Amsterdam, Amsterdam, The Netherlands; 3 Department of Pathology, VU University Medical Center, Cancer Center Amsterdam, Amsterdam, The Netherlands; 4 Department of Neurology, VU University Medical Center, Cancer Center Amsterdam, Amsterdam, The Netherlands; 5 Department of Pathology, Radboud University Medical Center, Nijmegen, The Netherlands; 6 Department of Neurology, Massachusetts General Hospital, Harvard Medical School, Boston, MA USA; 7 thromboDx BV, Amsterdam, The Netherlands

**Keywords:** Malignant glioma, Minimally invasive biomarkers, Liquid biopsies, Molecular diagnostics

## Abstract

Diffuse gliomas are the most common malignant primary tumors of the central nervous system. Like other neoplasms, these gliomas release molecular information into the circulation. Tumor-derived biomarkers include proteins, nucleic acids, and tumor-derived extracellular vesicles that accumulate in plasma, serum, blood platelets, urine and/or cerebrospinal fluid. Recently, also circulating tumor cells have been identified in the blood of glioma patients. Circulating molecules, vesicles, platelets, and cells may be useful as easily accessible diagnostic, prognostic and/or predictive biomarkers to guide patient management. Thereby, this approach may help to circumvent problems related to tumor heterogeneity and sampling error at the time of diagnosis. Also, liquid biopsies may allow for serial monitoring of treatment responses and of changes in the molecular characteristics of gliomas over time. In this review, we summarize the literature on blood-based biomarkers and their potential value for improving the management of patients with a diffuse glioma. Incorporation of the study of circulating molecular biomarkers in clinical trials is essential for further assessment of the potential of liquid biopsies in this context.

**Electronic supplementary material:**

The online version of this article (doi:10.1007/s00401-015-1399-y) contains supplementary material, which is available to authorized users.

## Introduction

Diffuse gliomas are the most frequent primary malignant tumors of the central nervous system [182]. Each year in the United States, approximately 17,000 patients are diagnosed with a diffuse glioma [119]. Nowadays, magnetic resonance (MR) imaging is the principal imaging modality for patients with suspected brain lesions [123]. For a definitive diagnosis of tumor type and malignancy grade, tumor tissue (obtained via biopsy or resection) is required [99]. Both low- and high-grade malignant diffuse gliomas are characterized by extensive, infiltrative growth in the brain parenchyma, rendering complete resection of the tumor impossible [36]. Even with the current standard treatment consisting of (combinations of) surgery, temozolomide (TMZ)—or procarbazine–lomustine–vincristine (PCV)-based chemotherapy—and radiotherapy [159, 160], glioma patients still face a poor prognosis. In general, the median survival of low-grade glioma (LGG; WHO grade II) patients is 5–10 years, 3 to more than 10 years for WHO grade III oligodendrogliomas, 2 years for WHO grade III astrocytomas, and 12–18 months for WHO grade IV gliomas/glioblastomas [133]. Bailey and Cushing proposed the first systematic and detailed classification of gliomas in 1926 [7]. In the course of the following decades, the morphological diagnosis (typing and grading) of these tumors was further refined, culminating in the most recent, i.e., 2007 World Health Organization (WHO) classification of diffuse gliomas [101]. Meanwhile, it is now fully clear that for optimal management of patients with a diffuse glioma more precise classification of these tumors is needed, and that molecular markers hold great promise in this respect [100, 108, 118, 170, 179, 180]. For instance, it has become clear that patients with grade III 1p/19q co-deleted anaplastic oligodendrogliomas generally have a better prognosis than patients with other grade III glioma subtypes and that *IDH1* mutant (secondary) glioblastomas tend to have a better outcome than *IDH1* wild-type grade III astrocytomas [164].

Biomarkers are defined here as objectively measurable parameters that have additional (diagnostic, prognostic, predictive and/or monitoring) value to clinical parameters such as tumor type and grade, age, and performance status [81, 109, 133]. Detection of molecular glioma biomarkers such as *MGMT* promoter methylation, *IDH1*/*IDH2* mutation, 1p/19q co-deletion, epidermal growth factor receptor (*EGFR*) amplification and EGFR variant III (EGFRvIII) expression in tumor tissue is increasingly being used in clinical care [64, 70, 183]. For example, 1p/19q co-deletion in diffuse gliomas serves as a diagnostic, prognostic, and predictive biomarker (indicating resp. the diagnosis of oligodendroglioma, improved survival and improved response to first-line PCV chemotherapy and radiation) [166]. New insights into gliomagenesis [22, 121, 169] such as the potential role of glioma stem-like cells [154], intratumoral heterogeneity [122, 156] and molecular subtypes [125, 161, 174] have revealed novel potential therapeutic targets. Examples are vascular endothelial growth factor (VEGF) which can be targeted by the monoclonal antibody bevacizumab, *IDH1*/*IDH2* mutations and the development of the specific inhibitor AGI-5198, and EGFRvIII with the peptide vaccine rindopepimut [6, 35, 57, 135]. Also, mutated *IDH1* has been reported as a marker indicating that more radical surgical glioma resection may be of benefit for the patient [14].

Non-small cell lung cancer and melanoma are at the forefront of molecular diagnostics, with a variety of treatments targeting molecular alterations defining specific cancer subtypes (e.g., *BRAF*, *EGFR*, *ALK* alterations). Accurate molecular diagnostics is essential for personalized therapies to assess patient candidacy for a particular therapy based on drug-sensitizing genetic alterations of the tumor. These specific genetic pathway alterations that are the target of a drug are regarded as molecular companion diagnostic biomarkers. The FDA has issued guidelines stating that they will only review such targeted drugs for approval in the context of corresponding in vitro companion diagnostics [146]. Companion diagnostics for targeted cancer treatments simplifies the drug discovery process, makes clinical trials more efficient and informative, and can be used to individualize the therapy of cancer patients. There is an urgent need to develop molecular diagnostics to better identify the patients that respond to expensive targeted therapies. Currently, mutational profiles of cancer patients are determined in tumor tissues. Examples are the Cobas 4800 BRAF V600 Mutation Test (Roche Molecular Systems Inc.) which detects the BRAF V600E mutation in formalin-fixed, paraffin-embedded (FFPE) human melanoma tissue and the Vysis ALK Break Apart FISH Probe Kit (Abbott Molecular Inc.), which detects rearrangements involving the *ALK* gene via fluorescence in situ hybridization (FISH) in FFPE tissue. However, limited access to serial tumor biopsies constitutes a major shortcoming in longitudinal molecular monitoring of targeted treatment. In addition, molecular diagnostics based on analysis of biopsied or surgically resected tumor tissue is hampered by sampling error. Many types of cancer, including melanoma, non-small cell lung cancer, and in particular glioma, have demonstrated to be of heterogeneous nature [156]. Glioma tissue heterogeneity may confound assessment of molecular markers such as EGFRvIII expression and *MGMT* promoter methylation. Such molecular diagnostic problems could be solved by minimally invasive techniques, possibly via advanced imaging modalities [9, 172] or ‘liquid biopsies’ [40, 66, 72, 88, 129, 137, 155, 168]. Additionally, liquid biopsies might allow for faster and more accurate tumor diagnosis of difficult-to-diagnose patients, e.g., patients presenting with a ring-enhancing lesion in the brain as measured by MR imaging that can be diagnosed as either a metastasized solid tumor or a primary brain tumor [26]. On top, molecular markers obtained via liquid biopsies may allow for repeated assessment of molecular aberrations of the tumor and real-time treatment monitoring in patients with diffuse glioma.

Importantly, whereas pathologists in conjunction with clinical geneticists are in the midst of a transformation towards molecular cancer diagnostics, the field of blood-based cancer analysis is still awaiting such a full transformation. Blood-based analysis, historically often positioned at the departments of clinical chemistry and microbiology, may likely benefit from a repositioning within the medical disciplines involved. In practice, the optimal development of diagnostics based on liquid biopsies—defined here as biofluids used for molecular diagnostics—will require next-level interdisciplinary action of clinical chemists, clinical geneticists, microbiologists, and (molecular) pathologists, among others. In this review on liquid biopsies in patients with diffuse glioma, we provide a concise discussion of the current state of the art of five blood-based liquid biopsy biosources [plasma; serum; extracellular vesicles; blood platelets (thrombocytes); circulating tumor cells (CTCs)], followed by a more elaborate discussion regarding the potential of detecting circulating proteins, circulating nucleic acids (DNA, different forms of RNA) and of circulating tumor cells.

## Biosources in liquid biopsies for molecular markers

**Fig. 1:**
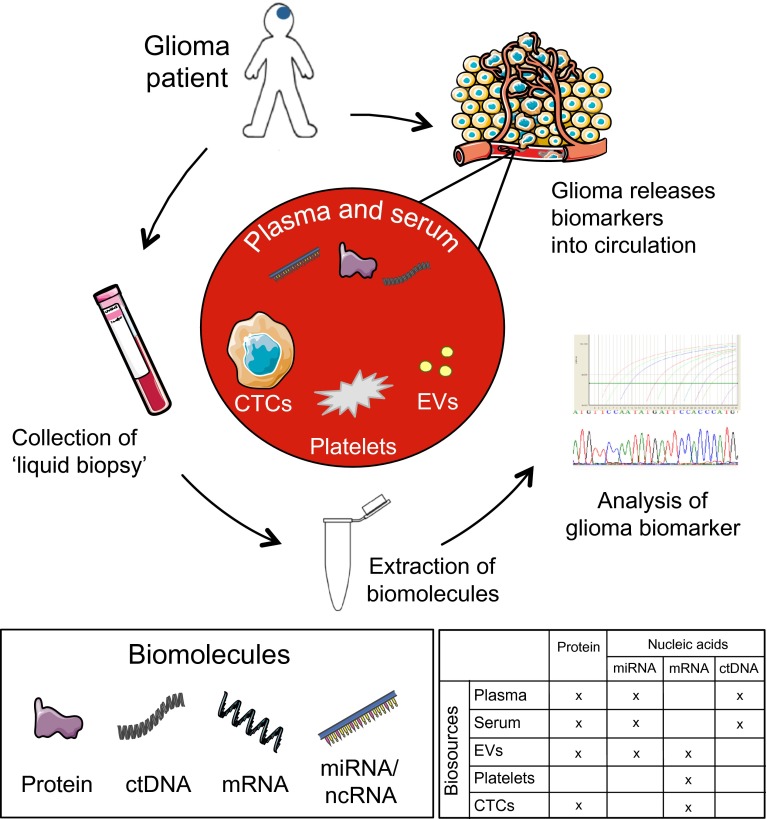
Overview of biomarker release, collection and assessment. In (glial) tumors, both neoplastic cells and the tumor-microenvironment release biomolecules in the circulation, either as circulating proteins (CP), circulating nucleic acids (CNA), or even complete circulating tumor cells (CTC). Blood plasma and serum are a source of circulating tumor DNA (ctDNA), circulating microRNAs (miRNAs) and circulating proteins. From whole blood, also extracellular vesicles (EVs), platelets (which internalize EVs) or complete CTCs can be isolated and used as potential biosources. These five biosources provide proteins, miRNAs, messenger RNAs (mRNA) non-coding RNAs (ncRNA) and ctDNA biomolecules (see separate lower right table). Of note, in the lower right table only biomolecules that were already identified in the particular biosources in patients with diffuse glioma are marked. These biomolecules and cells can be collected from a routinely obtained peripheral blood sample and used for further analyses in the laboratory

Plasma and serum are collected from whole blood and are the most studied biosources for molecular markers (Fig. 1). Circulating tumor DNA (ctDNA) and microRNAs (miRNAs) are relatively stable in plasma and serum, as opposed to messenger (mRNA) that is easily degraded. Nevertheless, within several hours of release ctDNA is removed from the circulation by the liver and the kidneys [149]. Plasma and serum also contain extracellular vesicles (EVs), which are small lipid membrane-bound satchels expelled by cells to mediate cell-to-cell communication and containing a wide collection of nucleic acids, lipids, and proteins [4, 43, 54, 155]. The physical structure of EVs protects nucleic acids from degradation by circulating nucleases [92, 155]. Indeed, isolation of RNA from serum-derived EVs from patients with glioblastoma can yield a 60× greater concentration of RNA compared to free-circulating RNA from whole blood, plasma or serum [30], which makes EVs a promising source for circulating RNA biomarkers. Complementary, circulating platelets are supplied by gliomas with tumor-derived EVs and contain tumor-derived RNA [116]. Interestingly, the majority of EVs circulating in blood are also derived from platelets, suggesting that platelets efficiently sequester and release EVs [134, 187]. Similar to EVs, internalized nucleic acids in platelets are protected from endogenous nucleases. Finally, CTCs, although widely studied for many solid tumors [87], were only recently detected in the blood of patients with glioma [104, 113, 162]. The origin of circulating biomarkers, either directly derived from glioma cells or indirectly released by cells in the tumor microenvironment, such as inflammatory, endothelial and stromal cells, or at more distant sites like the bone marrow, is not known for all molecular biomarkers. Obviously, EGFRvIII and IDH mutant proteins or nucleic acids are released primarily by glioma cells, whereas potential biomarkers such as VEGF can be derived from multiple cell types. Also, both active secretion and passive release are possible routes for biomolecules to enter the circulation, e.g., via apoptotic or necrotic tumor or tumor-infiltrating cells [40, 149]. In this review, cerebrospinal fluid (CSF) and urine are more marginally discussed as alternative biosources. As brain tumors are located intracranially and the brain is surrounded by CSF, this fluid may be an additional attractive liquid biopsy biosource for glioma [132]. Several studies have shown the suitability of detecting tumor-derived circulating nucleic acids [1, 10, 32, 132] and proteins [51, 53, 69, 83, 89, 90, 97, 115, 124, 142–144, 148, 153, 189, 191, 194] in CSF. However, collecting CSF is a more invasive, and burdensome procedure that harbors risks, particularly the risk of brain herniation in subjects with intracranial space-occupying lesions. Biomarkers can be either directly released into the CSF or accumulate via the brain interstitial fluid and perivascular drainage system into the cerebrospinal fluid [23, 41, 75]. Possibly, potential biomarkers in CSF also end up like a ‘sink effect’ in the circulating blood [92].

The individual candidate biomarkers are discussed below in order of biomarker class, i.e., circulating proteins (CPs) or circulating nucleic acids (CNAs), and further subclassified according to biomarker type (diagnostic, prognostic, predictive, monitoring). The biofluids, biosources, biomarker classes, and biomarker types suitable for liquid biopsies are summarized in Fig. 1. Moreover, we discuss current and future issues which may benefit from assessment of circulating biomarkers and lead to improved clinical care for patients with diffuse glioma.

## Circulating proteins (CPs)

Under several pathological conditions including cancer, the secretion, shedding and/or loss of proteins can become differentially regulated and subsequently result in increased or decreased levels of circulating proteins (CP) in blood, urine and/or CSF [17, 185]. The majority of studies concerning protein markers is performed on blood [number of studies (*n*) = 29 for plasma, and *n* = 40 for serum], although also CSF (*n* = 19) and urine (*n* = 4) have been reported as biosources for CPs in these patients as well. In blood-based glioma biomarker discovery studies, proteins are so far the most extensively studied biomolecules (summarized in Supplementary Table 1). The majority of these reports is of an exploratory nature and most molecules have not yet been evaluated in large clinical trials.

### Diagnostic circulating protein markers

First attempts to detect CP markers in blood of patients with glioma resulted predominantly in the identification of markers differentially expressed between patients and healthy controls. Plasma- and serum-derived protein markers—such as immunosuppressive acidic protein, alpha-1 acidic glycoprotein and alpha-1 antitrypsin, the glycoprotein fibronectin, and the endothelial cell-derived thrombomodulin-1—were the first proteins to be found elevated in the blood of patients with glioma [84, 110, 141, 145], followed by protein markers related to angiogenesis in gliomas. The serum and plasma protein levels of the marker VEGF have often been reported as significantly increased in patients with glioma compared to healthy controls [2, 28, 37, 128, 131], and even higher in patients with metastatic brain lesions [71, 128]. Soluble VEGFR-1 (sVEGFR-1), but not sVEGFR-2, -3, and basic fibroblast growth factor (bFGF, alternatively known as FGF-2), have been reported to be increased in pre-operatively collected serum samples from newly diagnosed glioblastoma patients [39, 131]. Also, markers involved in tumor cell invasion and remodeling of extracellular matrix, such as matrix metalloproteinases (MMPs) and tissue inhibitors of metalloproteinases (TIMPs), were identified as potential diagnostic CP biomarkers. Increased levels of MMP-2 and 9 were observed in CSF of high-grade glioma patients [51], and a larger study confirmed increased plasma levels of MMP-9 and TIMP-1 in patients with both grade II and IV glioma compared to healthy controls [95]. Surprisingly, however, in this study no difference was observed in the plasma protein levels of MMP-9 and TIMP-1 of grade III glioma patients as compared to healthy controls. Serum and plasma levels of TIMP-1 allowed the discrimination of grade IV from lower grade gliomas, and were confirmed in two additional studies [39, 95, 155, 157]. Furthermore, glial fibrillary acidic protein (GFAP) was suggested to have diagnostic value [24, 68, 73, 80, 102]. An increase of circulating GFAP levels was observed in pre-operatively collected samples from grade III and IV glioma patients in both serum and plasma [68, 73]. Also in a subset of patients with oligodendroglial tumors, high levels of GFAP were found in plasma, implying that circulating GFAP is not specific for high-grade malignant astrocytic neoplasms [68]. Of interest, in patients with multiple sclerosis or a metastatic brain lesion, plasma GFAP levels were found to be within the reference range [73, 102]. Plasma GFAP may, together with plasma placental growth factor (PlGF), differentiate between radiologically suspected high-grade glioma and brain metastases [73]. Obviously, the fact that not only GFAP, but also other markers such as brain-derived neurotrophic factor (BDNF) and S100 calcium binding protein B (S100B), can be detected in the circulation of healthy controls negatively influences the specificity of such protein markers for detection of disease processes [91]. Finally, urine protein levels of 2-hydroxyglutarate (2-HG) may aid in distinguishing *IDH1* mutant glioma patients from those with *IDH1* wild-type glioma [98]. Assessment of the 2-HG metabolite in pre-operatively collected serum samples did not allow for such discrimination [27]. Potential diagnostic value has also been reported for serum and/or plasma levels of EGFR protein [127, 151], transforming growth factor beta (TGFb) [147], tumor necrosis factor alpha (TNFa) [2, 28, 59, 131], interleukin 2 (IL-2) and its receptor [59, 192], Chitinase-3-like protein 1 (CHI3L1, also known as YKL-40) [15, 167], S100B [73, 102, 175], neural cell adhesion molecule (NCAM) [171], neuropeptide Y (NPY) [73] and BDNF [73].

### Prognostic circulating protein biomarkers

CP markers with prognostic value can be subdivided in two subgroups; tumor-related markers and markers associated with endogenous systemic stress responses. The glioma-related serum and plasma markers YKL-40, osteopontin (OPN) and the extracellular domain of EGFR were described to be inversely correlated with overall survival [15, 63, 67, 77, 127, 157]. Interestingly, increased serum levels of YKL-40 1 week after surgery and during follow-up of glioma patients, both newly diagnosed and recurrent, were associated with increased risk of death, also when adjusted for age, Karnofsky performance scale (KPS) and extent of surgical resection [15, 67, 77]. Furthermore, serum levels of YKL-40 appeared to be relatively stable over time [167]. In patients with recurrent glioblastoma prior to treatment with a combination of the monoclonal antibody bevacizumab and the topoisomerase 1 inhibitor irinotecan, elevated plasma levels of MMP-2 and decreased plasma levels of MMP-9 were associated with increased overall survival (OS) [165]. In addition, many CP markers implicated in tumor angiogenesis are associated with the survival of patients with glioma. The plasma levels of IGFBP-2 and VEGF, the serum level of plasminogen activator inhibitor-1 (PAI-1), and the CSF protein levels of hepatocyte growth factor (HGF), bFGF and VEGF were inversely associated with progression-free survival (PFS) and OS [49, 76, 94, 124, 188]. Elevated levels of these CP markers may reflect more aggressive tumors with extensive angiogenesis and subsequent leakage of tumor-derived molecules into the circulation. Furthermore, a stress response of the body, represented by elevated levels of C-reactive protein (CRP), the S100B protein, d-dimer and several heat shock proteins, has been associated with worse prognosis [65, 110, 158, 175]. Altogether, the most promising prognostic CP marker for glioma patients thus far seems to be serum YKL-40 protein.

### Predictive circulating protein markers

Predictive CP markers in patients with glioma may facilitate therapy selection without the need for invasive tumor tissue biopsies. So far, predictive CP markers for patients with diffuse glioma were mainly identified in phase I and II clinical trials testing the effect of anti-angiogenic treatments (esp. bevacizumab). Nevertheless, also for other established therapy regimens in patients with glioma, like TMZ treatment (see also CNAs), predictive CP markers might contribute to patient stratification. Tabouret et al. [165] reported that increased plasma protein levels of MMP-2 (>227.5 ng/ml) prior to combinatorial bevacizumab and irinotecan treatment were associated with higher probability of response to treatment (83 % compared to 15 %). Decreased plasma protein levels of MMP-9 (<235 ng/ml) were considered to be related to treatment response, although this was not confirmed in an additional patient cohort [165]. In a relatively small study (*n* = 10 recurrent glioblastoma patients), Chinnaiyan et al. [34] identified IGFBP-5 as a possible predictive protein marker for combined treatment with bevacizumab, HDAC inhibitor vorinostat, and irinotecan. Finally, non-responders to a combination therapy with bevacizumab and irinotecan had significantly increased plasma levels of IL-8 and G-CSF prior to start of the treatment as compared to responders [46].

### Monitoring circulating protein markers

Circulating protein markers in patients with glioma that can be potentially employed to monitor the efficacy of a particular treatment are gaining attention. The individual biological tumor properties and complex tissue alterations induced by different anti-tumor treatments are not properly assessed with current MR imaging protocols and response criteria [181]. These criteria consider in particular two of the imaging characteristics: contrast uptake and alteration of the T2-weighted signal. Both characteristics, however, do not always properly reflect tumor behavior. Contrast enhancement indicates disruption of the blood–brain barrier related to tumor progression, but anti-angiogenic treatment reduces the number and permeability of tumor blood vessels and decreases the amount of MR contrast agent in the tumor, resulting in potential misinterpretation of tumor regression (pseudo-response), whereas combined chemoirradiation may lead to temporarily increased contrast enhancement, erroneously interpreted as tumor progression (pseudo-progression). T2-weighted signal changes suggest diffuse infiltrative tumor progression, but might also be caused by tumor- or treatment-induced edema, resulting in misinterpretation as progression [163].

Blood-based biomarkers may allow circumvention of problematic pseudo-responses upon anti-angiogenesis treatment and pseudo-progression upon chemoradiotherapy treatment. Two studies that together included approximately 90 recurrent glioblastoma patients treated with bevacizumab plus irinotecan demonstrated that a decreased plasma protein level of VEGF, measured, respectively, eight weeks and 15 days after the start of the treatment, was associated with improved PFS and OS [46, 165]. In addition, decreased plasma protein levels of MMP-9 were associated with prolonged PFS and OS, whereas changes in the plasma protein levels of bFGF, SDF-1a, PlGF or VEGFR-2 were not associated with the effect of bevacizumab [165]. Unfortunately, baseline levels of VEGF was not associated with PFS in the larger AVAglio study (*n* = 571) [139]. Treatment responses of recurrent glioblastoma patients (*n* = 26) treated with the VEGF and PlGF sequestering drug aflibercept were not associated with significant changes in plasma protein levels of VEGF, despite initial reduction of this marker in plasma and subsequent increases of circulating VEGF and PlGF after 2 weeks of treatment [62]. Nevertheless, increased expression of MMP-9 and certain chemokines (e.g., MCTP3, MIF, IP-10) was correlated with tumor progression after 28 days of treatment, and may serve as putative CP monitoring markers for aflibercept treatment.

Multiple studies assessing the efficacy of the pan-VEGFR RTK inhibitor cediranib in both newly diagnosed and recurrent glioblastoma patients have included circulating biomarker discovery as an important component of the clinical trials. CPs were assessed in plasma and urine samples at different time points during treatment. Most notably, in recurrent glioblastoma patients, increased plasma protein levels of PlGF and IL-8 and decreased levels of bFGF and sTie-2 proteins were associated with partial radiological responses, whereas increased plasma protein levels of sVEGFR-1, sTie-2 and SDF-1a and decreased levels of PlGF correlated with radiological progression. Furthermore, a trend was observed between bFGF and SDF-1a levels, tumor vessel size and tumor progression. Increased plasma levels of PlGF and bFGF measured one day after start of treatment for recurrent glioblastoma correlated with improved OS. Increased plasma levels of MMP-2 and urine levels of MMP-9 measured within 1 day after the start of treatment were associated with shorter OS and PFS, respectively [11, 13]. In a trial assessing the efficacy of cediranib combined with standard treatment in newly diagnosed glioblastoma patients, increased plasma protein levels of sVEGFR-1 and IL-8, after, respectively, 29 and 48 days of treatment, were associated with reduced PFS [12]. MMP-9 and sVEGFR-1 were identified in plasma as potential CP monitoring markers for a combination therapy of TMZ plus the VEGF and PDGFR inhibitor vatalanib [56].

## Circulating nucleic acids (CNA)

Tumor-associated CNAs have been identified in the blood of patients with several types of cancer as early as 1977 [50, 93], and may be suitable for diagnosis and monitoring of tumor treatment [44, 114]. Several classes of CNAs are detected; ctDNA including methylated DNA, mRNA, and small RNAs including miRNAs and transfer RNAs (tRNAs) (Fig. 1). Tumor-derived nucleic acids can be detected as cell-free entities, attached to lipid or protein structures, or as content of circulating EVs or blood platelets [38, 40, 43, 116, 150, 155]. Of note, in a large study in which the presence of detectable ctDNA in several types of cancer was assessed only in a minority (10 %) of patients with glioma ctDNA was detected [16], indicating that the use of this biomarker in this patient category is challenging. In this section, we summarize the literature on CNAs in patients with diffuse glioma, while Supplementary Table 2 provides a comprehensive overview of relevant studies on this topic.

### Diagnostic circulating nucleic acid biomarkers

So far, most diagnostic CNA markers in patients with glioma were detected by miRNA profiling. Circulating miRNAs exhibit great stability in plasma and serum [33, 111], and their involvement in glioma pathogenesis has been extensively studied [112]. Several groups reported that differential expression of multiple miRNA sets in plasma, serum and blood cells distinguishes glioma patients from healthy controls [74, 103, 136, 152, 178, 190]. Unfortunately, the overlap between studies regarding plasma miRNA biomarkers is limited, and further validation is needed. Two studies compared the content of small RNAs in serum-derived EVs from patients with glioblastoma and healthy controls. Interestingly, RNA isolated from EVs appeared to be enriched for small RNAs in both healthy individuals and glioblastoma patients [117]. Based on the small RNA content, patients with glioblastoma could be distinguished from healthy controls [106]. Of note, it was recently reported that miRNAs are post-transcriptionally modified by the enzymatic addition of nucleotides at the 3′-end of the mature miRNA, thereby affecting their release by tumor cells [86]. Hence, for miRNA biomarker studies in liquid biopsies it is of crucial importance to take into account the presence of such non-templated nucleotide additions, possibly changing the CNA biomarker landscape. Other serum CNA markers such as loss of heterozygosity (LOH) of chromosome arms 1p, 19q and 10q, and methylation of *p16/INK4a* and *RASSF1A* were also associated with the ability to identify patients with particular types of brain tumors [92, 105, 176]. Additionally, measurements of methylation of circulating ALU repeat sequences (i.e., non-coding transposable elements integrated in the human genome) in plasma demonstrated a significant correlation with tissue methylation and were reported to allow for distinguishing low- from high-grade glioma patients and healthy controls [31].

### Prognostic circulating nucleic acid biomarkers

The number of prognostic CNA biomarkers identified in patients with diffuse glioma so far is limited. Liu et al. [96] identified an association between hypermethylation of both *MGMT* and thrombospondin-1 in plasma of grade II and grade IV glioma patients and, counter-intuitively, an inferior OS. However, the authors do not provide detailed information on treatment regimens that may well have had an impact on the outcome of the included patients. In contrast, in a prospective study collecting longitudinal plasma samples from grade II–IV glioma patients, presence of methylated *MGMT* was associated with increased PFS and OS [48]. Finally, both increased plasma levels of miR-454-3p and hypomethylation of ALU repeat elements in serum were reported to be associated with worse prognosis [31, 152].

### Predictive circulating nucleic acid biomarkers

Predictive properties of CNA biomarkers were predominantly studied for *MGMT* promoter methylation, *EGFRvIII* and the *IDH1 R132H* mutation. In this respect, *EGFRvIII* and *IDH1 R132H* are regarded as companion diagnostic biomarkers. Patients with methylated *MGMT* detected in serum ctDNA showed a significantly higher response to BCNU treatment, measured by imaging and clinical examination, compared to non-methylated *MGMT* ctDNA [8]. However, methylated *MGMT* in serum did not predict treatment response to the combination therapy of TMZ and cisplatin, possibly due to the inhibitory effect of cisplatin on the MGMT enzyme [8, 21, 177]. Of note, these authors demonstrated a sensitivity of ~50–80 % and a specificity of 100 % of methylated *MGMT* in serum and plasma compared to the result of *MGMT* analysis of the tumor tissue (Supplementary Table 3). Detection of specific mutations and deletion variants is of interest for integration of blood-based markers in clinical trials, e.g., for the EGFRvIII-targeted immunotherapy using rindopepimut (CDX-110). Rindopepimut is currently being evaluated in a phase III study for newly diagnosed glioblastoma patients (ACT IV) and in a phase II study for recurrent glioblastoma patients in combination with GM-CSF (ReACT) [6]. Recently, EGFRvIII RNA has been detected in serum EVs of glioma patients [155] and *EGFRvIII* ctDNA in plasma [140]. Moreover, Nilsson et al. [116] documented efficient uptake of EVs by blood platelets and detected EGFRvIII mRNA in these elements. Based on this information, diagnosis of EGFRvIII through liquid biopsies could allow for patient selection for specific anti-EGFRvIII treatment during clinical follow-up and possibly also for treatment monitoring. In addition, blood-based diagnosis of EGFRvIII may help to circumvent the possible underdiagnosis of heterogeneously expressed EGFRvIII in tumor tissue. Another genetic event of particular interest for use in companion diagnostics is the *IDH1* *R132H* mutation [121, 135]. Although the *IDH1* mutation was detected in CSF EVs of glioblastoma patients, it could not be detected in serum EVs of the same patients [30, 32]. Boissellier et al. [19] detected mutant *IDH1* ctDNA with a sensitivity of 60 % and a specificity of 100 %. In this study, detection of mutant *IDH1* in LGG patients depended on tumor volume and in high-grade glioma patients on the presence of contrast enhancement as visualized by MR imaging.

### Monitoring circulating nucleic acid biomarkers

*EGFRvIII* ctDNA, or EGFRvIII mRNA in EVs or platelets may be considered as a monitoring marker in studies of therapeutics specifically or indirectly targeting EGFRvIII. The loss of ctDNA EGFRvIII was noted in two patients following gross total resection of the tumor [140]. Furthermore, assessment of the *MGMT* methylation status in blood during TMZ treatment revealed that the cumulative incidence of unmethylated *MGMT* promoter, measured in longitudinal samples from 58 patients with diffuse glioma, increased from 58 % at the start of TMZ treatment to 92 % after 12 months of follow-up. This may be explained by the selection and outgrowth of tumor subclones with unmethylated *MGMT* promoter during TMZ treatment [48]. Circulating miRNA markers, which raised to reference levels of healthy controls following surgery and radio-chemotherapy of patients with diffuse glioma, may also have monitoring value [178, 190]. Furthermore, the significantly higher levels of miR-23, -150, -197 and -548b-5p were reported to occur in serum of patients with radiation necrosis/astrogliosis (*n* = 11) rather than in patients with diffuse glioma and may be of value to distinguish tumor progression from pseudo-progression after radiation (AUC: 0.95 95 % CI 0.902–0.998) [190].

## Circulating tumor cells (CTCs)

Circulating tumor cells are neoplastic cells that are detected in the circulation, and that can potentially be used as biomarker biosource [120]. CTCs are isolated through sophisticated techniques that separate CTCs from other blood cells by their unique physical and biological properties [3]. While previous attempts to isolate glioma CTCs were unsuccessful [18, 107], three independent research groups recently reported isolation of CTCs from glioma patients using three different novel isolation and detection methods (see also Supplemental Table 2). First, fluorescence immunocytochemistry was performed on purified mononuclear cells for GFAP-positive, CD45-negative CTCs. In 28 out of 141 glioblastoma patients, CTCs were identified, while these CTCs were not detected in patients with brain metastases. Following surgery, no significant difference was observed in CTC counts before, during or after surgery and no correlation between CTC counts and clinical outcome was found [113]. Second, a telomerase-responsive adenoviral vector encoding a fluorescent reporter was employed to detect hTERT-positive cells, resulting in detection of CTCs in 8 out of 11 glioblastoma patients analyzed before radiotherapy and TMZ treatment. In two patients, the quantitation of CTCs allowed the separation of pseudo-progression from tumor progression as measured by MR imaging [104]. Finally, Sullivan et al. applied a CTC-iChip, which allows the removal of anucleated cells and depletion of leukocytes from the remaining cell pellet via magnetically tagged antibodies (CD16, CD45), yielding untagged and un-manipulated CTCs. Next, fluorescent probes targeting five markers for glioma cells (termed STEAM: *Sox2, Tubulin beta*-*3*, *EGFR*, *A2B5*, *c*-*Met*) were introduced. In 13 out of 33 glioblastoma patients, CTCs were detected. CTCs were enriched for transcripts associated with a mesenchymal glioblastoma signature. Interestingly, the expression levels of these transcripts were not always in accordance with the signature that was detected in the tissue of the corresponding glial tumor, suggesting a preference of a particular subset of tumor cells (not necessarily being the predominant tumor cell population) for entering the circulation. Moreover, no additional correlations between CTCs and clinical parameters were observed [162]. Of note, a potential role for circulating endothelial (precursor) cells and T-lymphocytes (esp. T-regulatory cells) as diagnostic, prognostic, predictive, and monitoring markers in patients with diffuse glioma has been suggested [5, 13, 37, 42, 47, 60, 61, 128, 130, 184, 193]. In addition, immune cells release a broad range of CPs that could be of interest in this respect. The diagnostic value of immune cell-derived CNAs remains to be investigated [187]. In conclusion, successful isolation of CTCs from the blood of glioma patients was demonstrated using different techniques. In 20–73 % of high-grade glioma patients CTCs were detected. It remains to be evaluated whether glioma CTCs fully represent the heterogeneous tumor cell population, and what the diagnostic, prognostic, predictive and monitoring potential of CTCs is in patients with diffuse gliomas.

## Conclusions and future directions

**Fig. 2:**
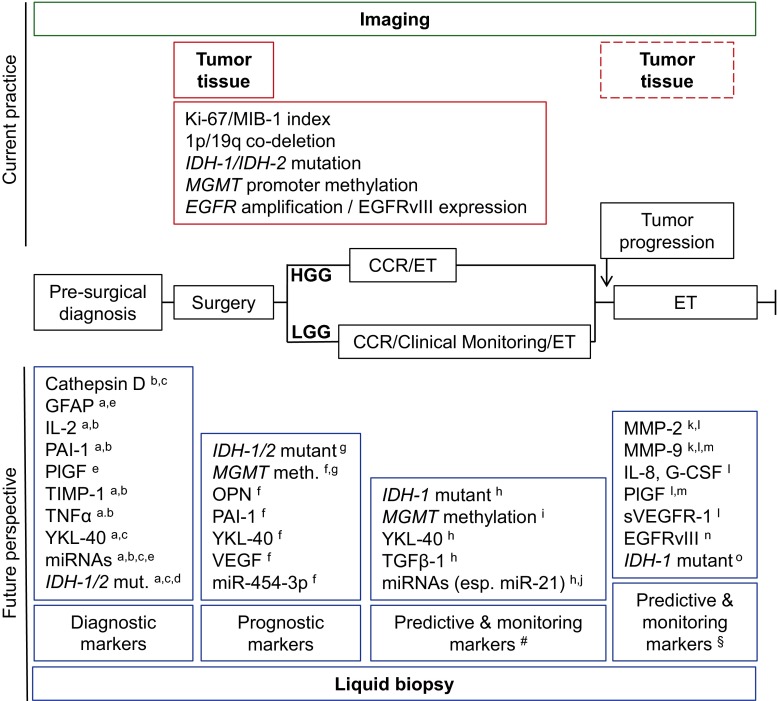
Example of molecular biomarker assessment using liquid biopsy in patients with low- and high-grade glioma. *Central black line* depicts a simplified timeline of clinical events for patients with low- or high-grade glioma (LGG versus HGG). A pre-surgical working diagnosis (based on esp. clinical and radiological investigations) leads to surgery (biopsy or resection of tumor tissue). After a pathological diagnosis is made, most glioma patients receive ‘conventional chemo- and/or radiotherapy’ (CCR), but in LGG patients clinical monitoring is also (still) common practice. Especially after failure of CCR, experimental therapy (ET) can be administered. Above the timeline, imaging (available during the entire follow-up of the patient) and the most widely accepted molecular biomarkers analyzed in diffuse glioma tissue samples are depicted. Tumor tissue samples are obtained during surgery, and might again be obtained at the moment of tumor progression (*dashed line box*). The *boxes* below the timeline show promising blood-based biomarkers as deduced from this review and grouped according to their potential meaning (diagnostic, prognostic, predictive, monitoring) in different phases of the disease process. The blood-based biomarkers mentioned here are selected based on the number of studies measuring the biomarker and the number of patients included in these studies. *Each character* indicates for which group of diffuse glioma patients or treatment the biomarker might be suitable. ^#^Predictive and monitoring markers selected for CCR regimens (temozolomide and procarbazine–lomustine–vincristine treatment). ^§^Predictive and monitoring markers selected for experimental therapies (see also individual characters). Characters accompanying biomarkers: *a* primary GBM (pGBM) vs. healthy controls, *b* pGBM vs. LGG, *c* LGG vs. healthy control, *d* secondary GBM vs pGBM, *e* primary diffuse glioma vs. metastasis to the brain, *f* HGG, *g* LGG, *h* surgery, *i* temozolomide treatment, *j* chemoradiotherapy, *k* bevacizumab and irinotecan treatment, *l* cediranib treatment, *m* aflibercept treatment, *n* rindopepimut (anti-EGFRvIII) treatment, *o* *IDH* *1* mutant inhibitor AGI-5198

Biomarkers can be isolated from biofluids such as blood, and also CSF and urine. There is a plethora of small and mainly explorative studies describing blood-based biomarkers in patients with diffuse glioma. The use of different techniques and methodologies for detection of the biomarkers and the mainly retrospective nature of the studies complicates drawing solid conclusions. In Fig. 2, we provide an overview of the biomarkers in liquid biopsies reported to be of potential relevance for diagnosis and/or management of patients with diffuse glioma. All blood-based biomarkers reported until now need further validation. Meanwhile, in the near future assessment of blood-based biomarkers can be expected to improve patient stratification and monitoring. Ideally, phase I trials should already include biomarker discovery, with further validation of potential biomarkers in phase II and III trials, including determination of proper cutoff values for biomarkers [77]. Furthermore, additional studies are needed that uncover potential circulating biomarkers accompanying MR imaging during clinical follow-up for, e.g., LGG patients. Finally, markers suggested in this review such as PlGF and GFAP have to be evaluated for its diagnostic potential for difficult-to-diagnose patients (e.g., in the differential diagnosis of metastasis versus primary brain tumor). The evaluation of biomarker panels [39, 45, 151], instead of sole biomarkers, using standardized detection methods, could be useful for further enhancing the sensitivity and specificity of blood tests [167]. This could be accompanied by a two or more steps decision tree algorithm as was recently shown for three diagnostic CP markers (GFAP, IGFBP-2, and YKL-40) [52]. The use of “omic-approaches” is likely to boost such a biomarker panel analysis [20, 55, 82, 83, 89, 97, 115, 148, 194].

The most extensively studied CP biomarkers are circulating angiogenesis-related proteins. While such proteins may indeed be useful as prognostic and monitoring biomarkers for anti-angiogenic therapies, a robust biomarker for the monitoring of bevacizumab treatment (i.e., the most widely applied anti-angiogenic therapy to date) has not yet been reported. Additionally, the discovery of tumor-derived ctDNA, circulating miRNAs, EVs and platelets sequestering tumor-derived EGFRvIII do point at the possibility to use blood as a source of CNA biomarkers in patients with diffuse glioma. An advantage of CNA biomarkers is the possibility to detect genetic aberrations such as *IDH1* mutations and *EGFRvIII*, which are of special interest as predictive markers for current clinical trials. Acknowledging that ctDNA so far was measurable in only a minority of patients with a diffuse glioma, detection of RNA might be more sensitive as compared to ctDNA, especially when analyzing the fraction of EVs or blood platelet in which RNAs are protected from circulating nucleases [16]. Assessment of circulating biomarkers may be of particular value for molecular markers that are known to be heterogeneously distributed (with a danger of sampling error) such as *EGFRvIII*. Also, *IDH1* mutations detected in ctDNA and EVs isolated from CSF may provide a way for non-invasive assessment of diagnostic and prognostic properties.

Thus far, many different tumor-related and tumor-derived markers have been identified in the circulation of patients with glioma that might attribute as specific biomarkers. Rational selection of the most promising biomarkers for incorporation in clinical trials is warranted, with literature mining as an important tool for such a selection. Potential biomarkers extracted from the present review of the literature for different stages of clinical management of patients with diffuse gliomas are summarized in Fig. 2. Obviously, regular and systematic evaluation of promising blood-based biomarkers, preferable on an international level via research communities such as the EORTC, would improve the assessment of their clinical value. Systematic collection of blood samples for future biomarker discovery and the establishment of blood biobanks, also outside clinical trails, would be very helpful in this respect. Ideally, such samples would be collected both prior to treatment (to allow for identification of diagnostic, prognostic and predictive biomarkers) as well as during therapy, the latter in particular situations in which robust predictive and monitoring biomarkers are still lacking (e.g., bevacizumab treatment).

Blood-based liquid biopsies are of interest for the longitudinal monitoring of tumor activity and therapy response. Depending on the blood fraction (i.e., serum or plasma), levels of CPs, CNAs, and possibly CTCs may vary considerably. Some markers have demonstrated biomarker value in plasma, whereas others were only of potential value in serum [188]. Also, used assays and methodologies for detection of CPs, (modified) CNAs and CTCs, each with its own strengths and weaknesses, have to be critically considered, and standardization is required to be able to compare results between studies. Furthermore, storage conditions of liquid biopsy samples require consideration, as well as other variables such as disease state, histological tumor subtype, time of sampling, biosource and biomarker isolation procedures, treatment regimen, assay reproducibility and sensitivity, protein half-life, biological processes, and normalization standards [25, 29, 39, 58, 73, 78, 79, 138, 147, 153, 173, 186, 195]. Of note, the sequestration of several proteins and nucleic acids in platelets and the potential release of proteins and nucleic acids by platelets and immune cells during surgery and blood processing have to be taken into account [39, 85, 147]. Therefore, to thoroughly address these issues and to control for them, large standardized clinical studies, requiring specific study designs and well-validated molecular assays, will be of critical importance. Importantly, proper selection of a control group with a disease that is a meaningful differential diagnosis for glioma patients (e.g., tumor metastasis to the brain, primary CNS lymphoma, or infection) is important, especially in biomarker discovery [52, 126]. Conventional biomarker discovery using state-of-the-art next-generation DNA/RNA sequencing or protein quantifications can be expected to yield novel biomarkers correlated to a specific disease state.

In conclusion, the interest in liquid biopsies for the diagnostics of glioma is rapidly expanding. In recent years, many circulating biomarkers have been detected, evaluated and integrated in clinical trials. In the coming years, more large-scale biomarker discovery efforts in patients with diffuse gliomas can be expected. Advances in the development of more sensitive assays allow for detection of low abundant (mutant) biomolecules in the circulation, further expanding the width of assessable biomarkers. Selection and validation of potential circulating biomarkers for patients with diffuse glioma is warranted. In the future, blood-based companion diagnostics may improve the drug discovery process for diffuse glioma patients and further individualize the therapy of these patients. Within the coming decade, the ongoing research may well result in an interdisciplinary transformation of the classical blood-based analyses towards the routine use of liquid biopsies for molecular diagnostics, similarly as we observed for tissue biopsies.

## Electronic supplementary material

Below is the link to the electronic supplementary material.

Supplementary material 1 (DOCX 186 kb)
Supplemental Table 1. Comprehensive overview of circulating protein markers in patients with diffuse glioma. Abbreviations: AA = Anaplastic Astrocytoma, AOD = Anaplastic Oligodendroglioma, CR = Complete Response, CNS = Central Nervous System, CP = Circulating Protein, CRT = Chemoradiotherapy, CSF = Cerebrospinal Fluid, DA = Diffuse Astrocytoma, DFS = Disease Free Survival, OD = Oligodendroglioma, ELISA = Enzyme-Linked Immunosorbent Assay, HGA = High-Grade Astrocytoma, HGG = High-Grade Glioma (n, r is newly-diagnosed and recurrent resp.), LGA = Low-Grade Astrocytoma, LGG = Low-Grade Glioma, MBL = Metastatic Brain Lesion, MR = Minor Response, MRI = Magnetic Resonance Imaging, MS = Multiple Sclerosis, nGBM = Newly diagnosed glioblastoma, OS = Overall Survival, PD = Progressive Disease, PFS = Progression Free Survival, PR = Partial Response, RT = radiotherapy, SD = Stable Disease, Sens = Sensitivity, Spec = Specificity, TTP = Time-to-Progression, WB = Western Blot
Supplemental Table 2. Comprehensive overview of circulating nucleic acids and circulating tumor cells in patients with diffuse glioma. AUC = Area Under the Curve, BCNU = 1,3-bis(2- chloroethyl)-1-nitrosourea chemotherapy, CRT = Chemoradiotherapy, CSF = Cerebrospinal Fluid, CTC = Circulating Tumor Cells, ctDNA = Circulating tumor DNA, HGG = High-Grade Glioma, LGG = Low-Grade Glioma, MP = Microparticle, mRNA = messenger RNA, miRNA = microRNA, PFS = Progression Free Survival, OS = Overall Survival, VTE = Venous thromboembolism
Supplemental Table 3. Overview of sensitivity of circulating *MGMT* promoter methylation. Table describes the sensititivity of *MGMT* promoter methylation in serum. Depicted per study population and detection methods. • = Cave: small study population (n = 2). Abbreviations: A = Astrocytoma, AA = Anaplastic Astrocytoma, AO = Anaplastic Oligodendroglioma, AOA = Anaplastic Oligoastrocytoma, GBM = glioblastoma (p, s, r is primary, secondary, recurrent resp.), OA = oligoastrocytoma, OD = oligodendroglioma
Supplemental Table 4. Overview of discussed markers and their status as biomarkers. Overview of all selected potential circulating biomarkers shown in Figure 2 including their potential as diagnostic, prognostic, predictive or monitoring biomarker
