# Supplementary material for: Liquid biopsies in patients with diffuse glioma
Source: Acta Neuropathol. 2015 Feb 27;129(6):849–65. doi: 10.1007/s00401-015-1399-y (PMC4436687; doi:10.1007/s00401-015-1399-y)
Supplement: Supplementary file 1 — Supplementary material 1 (DOCX 186 kb) Supplemental Table 1. Comprehensive overview of circulating protein markers in patients with diffuse glioma. Abbreviations: AA = Anaplastic Astrocytoma, AOD = Anaplastic Oligodendroglioma, CR = Complete Response, CNS = Central Nervous System, CP = Circulating Protein, CRT = Chemoradiotherapy, CSF = Cerebrospinal Fluid, DA = Diffuse Astrocytoma, DFS = Disease Free Survival, OD = Oligodendroglioma, ELISA = Enzyme-Linked Immunosorbent Assay, HGA = High-Grade Astrocytoma, HGG = High-Grade Glioma (n, r is newly-diagnosed and recurrent resp.), LGA = Low-Grade Astrocytoma, LGG = Low-Grade Glioma, MBL = Metastatic Brain Lesion, MR = Minor Response, MRI = Magnetic Resonance Imaging, MS = Multiple Sclerosis, nGBM = Newly diagnosed glioblastoma, OS = Overall Survival, PD = Progressive Disease, PFS = Progression Free Survival, PR = Partial Response, RT = radiotherapy, SD = Stable Disease, Sens = Sensitivity, Spec = Specificity, TTP = Time-to-Progression, WB = Western Blot Supplemental Table 2. Comprehensive overview of circulating nucleic acids and circulating tumor cells in patients with diffuse glioma. AUC = Area Under the Curve, BCNU = 1,3-bis(2- chloroethyl)-1-nitrosourea chemotherapy, CRT = Chemoradiotherapy, CSF = Cerebrospinal Fluid, CTC = Circulating Tumor Cells, ctDNA = Circulating tumor DNA, HGG = High-Grade Glioma, LGG = Low-Grade Glioma, MP = Microparticle, mRNA = messenger RNA, miRNA = microRNA, PFS = Progression Free Survival, OS = Overall Survival, VTE = Venous thromboembolism Supplemental Table 3. Overview of sensitivity of circulating MGMT promoter methylation. Table describes the sensititivity of MGMT promoter methylation in serum. Depicted per study population and detection methods. • = Cave: small study population (n = 2). Abbreviations: A = Astrocytoma, AA = Anaplastic Astrocytoma, AO = Anaplastic Oligodendroglioma, AOA = Anaplastic Oligoastrocytoma, GBM = glioblastoma (p, s, r is primary, secondary, recurrent resp.), [file 401_2015_1399_MOESM1_ESM.docx]

**Supplemental table 1 – Comprehensive overview of circulating protein markers in patients with diffuse glioma**

| **Ref** | **Bio-**  **source** | **Assay** | **Marker**  **proteins** | **Population**  **size (n)** | | **Diagn** | **Progn** | **Pred** | **Monit** | **Patients** | **Sampling** | **Main conclusions** |
| --- | --- | --- | --- | --- | --- | --- | --- | --- | --- | --- | --- | --- |
|  |  |  |  | **Ctrl** | **Glioma** |  |  |  |  |  |  |  |
| 2. | Serum | Luminex (xMAP assay), ELISA | IL-1β,  IL-4, IL-6, IL-10, GM-CSF, TNFα,  bFGF, VEGF | 20 | 55 | x |  |  |  | Not specified | Not specified | Significantly lower (IL-4, IL-10,  GM-CSF) or higher (IL-1β, IL-6, TNFα, bFGF, VEGF) in patients with GBM as compared to controls. |
|  |  |  |  |  |  |  |  |  |  |  |  |  |
| 196. | Plasma | ELISA | Filamin-A | 15 | 49 | x |  |  |  | Not specified | Prior to surgery | Higher levels in GBM patients as compared to astrocytoma (WHO II and -III) patients and healthy controls. |
|  |  |  |  | 19 | 10 | x |  |  |  |  |  | Higher levels in oligodendroglioma (WHO II and -III) patients than in healthy controls. |
| 13. | Plasma | ELISA | PlGF, sVEGFR-2, bFGF, SDF-1a | - | 16 |  |  |  | x | Recurrent | During treatment (cediranib) | Increase (sVEGFR-2, bFGF, SDF-1a) or decrease (PlGF) associated with tumor progression/relapse in patients with GBM treated with cediranib. bFGF and SDF1a positively correlate with vessel size on MRI. |
| 11. | Plasma | ELISA | MMP-2 | - | 31 |  |  |  | x | Recurrent | During treatment (cediranib) | Increased MMP-2 levels after 8h of treatment associated with shorter OS and PFS of patients with GBM. |
|  |  |  | PlGF, bFGF | - | 31 |  |  |  | x | Recurrent | During treatment (cediranib) | Increases in PlGF & bFGF after 1 day of treatment associated with better OS of patients with GBM. |
|  |  |  | PlGF, IL-8, bFGF, sTie-2, sVEGFR-1, SDF-1a | - | 31 |  |  |  | x | Recurrent | During treatment (cediranib) | Higher (PlGF, IL-8) or lower (bFGF, sTie-2) levels associated with radiographic PR in patients with GBM. Higher levels of sVEGFR-1, sTie-2 and SDF-1a correlated to radiographic progression. |
|  | Urine | Gel zymo-graphy | MMP-9 | - | 31 |  |  |  | x | Recurrent | During treatment (cediranib) | Increased MMP-9 activity after 1 day of treatment associated with poor PFS of patients with GBM. |
| 12. | Plasma | ELISA | sVEGFR-1 | - | 40 |  |  |  | x | Newly diagnosed | During treatment (CRT + cediranib) | Higher levels of sVEGFR-1 after  29 days of treatment associated with shorter OS and PFS of patients with GBM. |
|  |  |  | IL-8, sVEGFR-2, PlGF | - | 40 |  |  |  | x | Newly diagnosed | During treatment (CRT + cediranib) | Higher levels of IL-8 after 43 days of treatment correlated with shorter PFS of patients with GBM. Increases in PlGF and decreases in sVEGFR2 associated with increased blood perfusion. |
| 15. | Serum | ELISA | YKL-40 | 20 | 60 | x |  |  |  | Newly diagnosed | Prior to surgery | Significantly higher pre-resection levels in patients with GBM as compared to healthy controls. |
|  |  |  |  | 20 | 60 |  |  |  | x | Newly diagnosed | After surgery | Higher levels in patients with GBM with subtotal resection as compared to total resection. Levels one week and one month post-resection correlated with the tumor size on MRI, measured after one month. |
|  |  |  |  | 20 | 60 |  | x |  |  | Newly diagnosed | Change from baseline | Higher ratio (level one week post-resection/pre-resection level) associated with shorter OS of patients with GBM. |
| 20. | CSF | Mass finger-printing, MS/MS, WB | Apolipo-protein  A-II | 70 | 11 | x |  |  |  | Newly diagnosed | Not specified | Present in 32 pediatric patients with brain tumors, absent in controls. |
| 24. | Serum | ELISA | GFAP | - | 31 | x |  |  |  | Newly diagnosed | Prior to surgery | Elevated in 16 HGG patients as compared to the normal reference range. However, no p-values mentioned. Significant association between GFAP and tumor size. |
| 28. | Plasma | Anti-body array (micro-array) | C5, IL-8, IL-12, VEGF, Factor B, IL-6, IL-9, IL-16, IFNy, TNFα | 17 | 18 | x |  |  |  | Not specified | Prior to surgery | Different CP levels between GBM patients and healthy controls. |
|  |  |  | IL-1β, IL-3, IL-9, IL-12, IL-13, IFNγ, TNFβ, Lewis Y, PSA, CD40 | - | 18 |  |  | x |  | Not specified | Prior to surgery | Different CP levels between GBM patients responding to immunotherapy and non-responders. |
|  |  |  | IL-1β, IL-6, IL-9, IL-12, IL-13, IL-16, CD40, VEGF, IFNγ, TNFα, TNFβ (and several more) | - | 8 |  |  |  | x | Not specified | During treatment (IFNy transfected glioma cells) | Increased CP levels in GBM patients responding to immunotherapy as compared to non-responders. |
| 34. | Plasma | Anti-body array | IGFBP-5 | - | 10 |  |  | x |  | Recurrent | Prior to treatment (vorinostat, beva-cizumab, irinotecan) | Lower than median levels predicts PFS (>6 months) in patients with GBM. |
|  |  |  | PDGF-AA | - | 10 |  |  |  | x | Recurrent | Change from baseline | Significantly lower levels in patients with GBM after recurrence as compared to pre-treatment (baseline). |
| 37. | Plasma | ELISA | VEGF | 34 | 11 | x |  |  |  | Not specified | Prior to surgery | Significantly higher levels in HGG patients compared to healthy controls. |
| 39. | Serum | Protein array | TIMP-1, TIMP-2, PDGF-AA, PDGFRα, Ang-1 | 5 | 36 | x |  |  |  | Newly diagnosed | Prior to surgery | Signficantly higher (TIMP-1, TIMP-2, PDGF-AA, Ang-1) or lower (PDGFRα) levels in patients with GBM as compared to controls. |
|  |  |  | TIMP-1 | - | 36 |  | x |  |  | Newly diagnosed | Prior to surgery | Negatively correlated to survival. GBM patients with higher levels of TIMP-1 have shorter OS. |
| 218. | Serum | ELISA | EGF | - | 56 |  | x |  |  | Recurrent | Prior to treatment (intranasal POH) | GBM patients with lower (<250 pg/ml) levels of EGF had longer OS compared to those with higher (>250 pg/ml) levels. No differences in circulating EGF levels between primary and secondary GBM patients. |
| 199. | Serum | ELISA | IL-6,  IL-17A | 26 | 38 | x |  |  |  | Not specified | Prior to surgery/  therapy | For IL-17A, significantly higher levels in glioma patients as compared to controls, patients with meningioma or -schwannoma. For IL-6, significantly lower levels in glioma patients vs. controls. |
| 45. | Serum | ELISA | RBP-4, Serotonin, SCF,  CXCL-10 | 12 | 23 | x |  |  |  | Not specified | Prior to surgery | Reduced (CXCL-10) or elevated (others) serum levels in GBM patients as compared to healthy controls. |
|  | Serum | ELISA | TSP-1, IGFBP-3 | - | 23 |  | x |  |  | Not specified | Prior to surgery | Significantly higher TSP-1 and  IGFBP-3 levels in patients with GBM with longer (>15 months) survival after surgery as compared to those with short (<15 months) survival. |
| 46. | Plasma | Bioplex sus-pension assay | IL-8,  G-CSF | - | 63 |  |  | x |  | Recurrent | Prior to treatment (beva-cizumab + irino-tecan) | Levels significantly higher in  non-responding HGG patients as compared to responders. |
|  |  |  | VEGF | - | 63 |  |  |  | x | Recurrent | Change from baseline | Significant decrease in VEGF level after 8 wks in a group of long-term (>18 weeks PFS) responding HGG patients. |
| 49. | Serum | ELISA | bFGF | - | 16 |  |  |  | x | Recurrent | Prior to and during treat-ment thalido-mide) | Increasing bFGF in HGG patients associated with radiographic progression, shorter TTP and -OS of patients, as oppossed to patients with stable or decreasing bFGF.  During treatment, HGG patients with clinically stable disease had stable or decreasing bFGF. |
|  |  |  |  |  |  |  |  |  |  |  |  |  |
| 51. | CSF | Gel zymo-graphy | Pro-  MMP-9,  activated MMP-9,  MMP-2 | 26 | 29 | x |  |  |  | Not specified | Not specified | Present in patients with brain tumors (glioma and metastatic cancer) and absent in controls. Also significantly higher activity of MMP-9 in brain tumor patients as compared to control. |
|  |  |  |  |  |  |  |  |  |  |  |  |  |
| 200. | Serum | ELISA | Cathep-sin-D | - | 20 | x |  |  |  | Not specified (≥6 newly diag-nosed) | Prior to treatment (CRT) | Significantly more HGG patients with levels >100 ng/mL as compared to patients with LGG. |
| 52. | Plasma | ELISA | GFAP,  YKL-40,  IGFBP-2 | 99 | 151 | x | x |  |  | 111 nGBM,  40 non glial brain tumors | Pre-operative | The combined levels of GFAP and YKL-40 discriminate GBM patients from other non-glial brain tumors. Level of IGFBP-2 was correlated with poorer PFS and OS. |
| 53. | CSF | ELISA | HGF | 25 | 14 | x |  |  |  | Not specified | Prior to surgery | Significantly higher levels in HGG- and meningioma patients compared to controls and in HGG patients as compared to patients with meningioma. |
|  | CSF | ELISA | HGF | - | 14 |  | x |  |  | Not specified | Prior to surgery | Negative correlation between HGF levels and OS of HGG patients (n=14). High concentrations (>500 pg/ml) associated with shorter OS. Furthermore, high levels (>850 pg/ml) in patients with GBM (n=10) associated with an increased rate of recurrence after gross total resection. |
|  |  |  |  |  |  |  |  |  |  |  |  |  |
| 55. | Plasma | LC-MS/MS (iTRAQ), ELISA | Set of 296 proteins | 3 | 3 | x |  |  |  | Not specified | Prior to surgery | Differentially expressed between patients with GBM and controls. |
|  |  |  | FTL, CNDP1, S100A9 | 10 | 10 | x |  |  |  | Not specified | Prior to surgery | Significantly elevated (FTL, S100A9) or reduced (CNDP1) in patients with GBM as compared to controls. |
| 56. | Plasma | ELISA | IL-1β, MMP-1, MMP-3, MMP-9, MMP-10, sVEGFR-1 | - | 17 |  | x |  |  | Newly diagnosed | Change from baseline | In- or decreased levels of markers compared to pre-treatment levels at multiple timepoints during treatment associated with OS of patients with GBM. |
|  |  |  | MMP-9, MMP-10 | - | 17 |  | x |  | x | Newly diagnosed | Prior to treatment (CRT + vatalanib) | Pre-treatment levels and levels after 1 day of treatment of MMP-9 associated with OS of patients with GBM. Also, levels of MMP-10 after four days of treatment associated with OS. |
|  |  |  | MMP-3 | - | 17 |  |  |  | x | Newly diagnosed | During treatment (CRT + vatalanib) | Levels after 70 days of therapy associated with the OS of patients with GBM. |
|  |  |  | MMP-9, MMP-10, SDF-1a | - | 17 |  |  |  | x | Newly diagnosed | Change from baseline | Levels of MMP-9 after 8 h, SDF-1a after 1 and 70 days, and MMP-10 after 9 days associated with PFS of patients with GBM. |
|  |  |  | Collagen IV,  MMP-2, MMP-10, SDF-1a | - | 17 |  |  |  | x | Newly diagnosed | During treatment (CRT + vatalanib) | Levels at several timepoints associated with PFS of patients with GBM. |
| 201. | Serum | WB | Hemo-poxin, cerulo-plasmin, plasma retinol binding protein | 40 | 40 | x |  |  |  | Newly diagnosed | Prior to surgery | Higher levels in GBM patients as compared to controls. Several other potential candidates identified by 2D-(DIGE)-MS/MS. |
|  | Serum | Immuno-turbidi-metric assay | Hapto-globin | 20 | 20 | x |  |  |  | Newly diagnosed | Prior to surgery | Higher levels in GBM patients as compared to controls. |
| 59. | Serum | Radial immuno-diffusion | C4 | 30 | 49 | x |  |  |  | Newly diagnosed | Prior to surgery | Reduced values in patients with GBM compared to controls. |
|  | Serum | ELISA | IL-2,  TNFα | 30 | 49 | x |  |  |  | Newly diagnosed | Prior to surgery | Significantly reduced values of IL-2 in patients with GBM compared to controls and signficantly higher levels of IL-2 and TNFα in LGG patients as compared HGG. IL-2 is a predictor for tumor grade. |
| 62. | Plasma | ELISA | PlGF | - | 26 |  |  | x |  | Recurrent | Prior to treatment (aflibercept) | Low levels in patients with GBM associated with response to aflibercept. |
|  |  | Multiplex sus-pension assay | CTACK, MCP-3, MIF, IP-10 | - | 26 |  |  | x |  | Recurrent | Prior to treatment (aflibercept) | High levels in patients with GBM  associated with response to aflibercept. |
|  |  | Multiplex sus-pension assay | TIMP-1 | - | 26 |  |  |  | x | Recurrent | Change from baseline  (log scale) | Increase at day 28 associated with risk of PD in GBM patients during treatment with aflibercept. |
|  |  | Linco-plex assay | MMP-9 | - | 26 |  |  |  | x | Recurrent | Change from baseline | Increase at day 28 correlated with tumor progression in GBM patients during treatment with aflibercept. |
| 202. | Serum (exo-somes & MVs) | WB | EGFRvIII, TGFb-1 | Not specified | 12 | x |  |  |  | Not specified | Prior to surgery | Present in EVs from HGG patients, absent in vesicles from normal serum. |
| 63. | Plasma | ELISA | OPN (total) | - | 33 |  |  |  | x | Not specified | After surgery, before and after RT | HGG patients with above median levels after RT had significantly shorter OS. Above median increases post-RT as compared to pre-RT also associated with worse OS. Glioma patients with larger tumor-, tumor necrosis volume prior to surgery and patients who underwent less invasive surgery had significantly higher levels pre-RT as well as post-RT. |
| 203. | Serum | Bioplex sus-pension assay | Angio-poietin, follistatin, HGF, IL-8, leptin, PDGF-BB, PECAM-1 | 25 | 50 | x |  |  |  | Newly diagnosed | Prior to surgery | Significantly higher (angiopoietin,  IL-8) or lower (follistatin, HGF leptin, PDGF-BB, PECAM-1) levels in patients with GBM as compared to controls. |
| 65. | Plasma | Quanti-tative latex assay | D-dimer | - | 23 |  | x |  |  | Not specified | Prior to treatment (RT/  CRT) | Elevated D-dimer levels (>1 μg/ml) associated with shorter OS and PFS of patients with GBM. |
| 67. | Serum | ELISA | YKL-40 | - | 143 |  | x |  |  | Mixed | Baseline (not specified, log scale) | Increase in YKL-40 associated with OS of GBM patients, not with OS of patients with WHO III glioma. |
|  | Serum | ELISA | YKL-40, MMP-9 | - | 143 |  |  |  | x | Mixed | During treatment (not specified) | YKL-40 levels associated with disease status of HGG patients. MMP-9 only with that of GBM patients. Patients with a CR had lower levels and the majority of GBM patients with a persistent CR had levels within the reference range. |
| 68. | Plasma | Electro-chemi-lumines-cent assay | GFAP | - | 23 | x |  |  |  | Newly diagnosed | Prior to surgery | Detected in HGG and OD (not in DA). Proportion of GFAP positive patients significantly higher in GBM as compared to WHO II and III gliomas. All GBM patients had detectable GFAP levels. |
| 69. | CSF | Quanti-tative immuno-blotting | Extra-cellular membrane particles | 14 | 61 | x | x |  |  | Mixed | Variable (marjority prior to surgery) | GBM patients with lower levels (corresponding to <7.4 ng antibody (Ab) bound to membrane-particle associated Prominin-1) have longer disease duration (>30 months) vs. those with higher levels (>7.4 ng Ab). Significantly higher in GBM patients as compared to controls. |
| 73. | Plasma | ELISA | S100b | 51 | 51 | x |  |  |  | Mixed | Prior to surgery/  RT | S100b only detectable in patients with HGG, absent in patients with WHO grade II glioma. |
|  |  | ELISA | GFAP | 51 | 51 | x |  |  |  | Mixed | Prior to surgery/  RT | GFAP is present in patients with GBM, in 2 out of 52 controls and absent from patients with WHO II, -III glioma or meningioma. |
|  |  | ELISA | BDNF, PlGF,  IL-8 | 51 | 51 | x |  |  |  | Mixed | Prior to surgery/  RT | Significantly higher (BDNF) or lower (PlGF, IL-8) in GBM patients vs. those with MBT. PlGF and IL-8 also differentiate patients with HGG from patients with MBL. |
|  |  | ELISA | IL-8 | 51 | 51 | x |  |  |  | Mixed | Prior to surgery/  RT | Significant inverse association with tumor grade. Higher in WHO II patients than in those with GBM or WHO III. |
|  |  | Radio-immuno-assay | NPY | 51 | 51 | x |  |  |  | Mixed | Prior to surgery/  RT | Significant inverse association with tumor grade. Higher in WHO II patients than in those with GBM. |
|  |  |  |  |  |  |  |  |  |  |  |  |  |
| 71. | Plasma | ELISA | Ang-2, VEGF | - | 34 | x |  |  |  | Not specified | Prior to surgery/  therapy | For Ang-2, significantly lower levels in glioma patients as compared to patients with meningioma and in patients with GBM vs. MBL patients. For VEGF, lower levels in patients with GBM as compared to MBL patients. VEGF was also elevated in patients in patients with multiple (>3) brain metastases vs. those with 1-3 lesions. |
| 204. | Serum | ELISA | APRIL, CRP | 25 | 25 | x |  |  |  | Not specified | Not specified | Significantly higher in patients with GBM as compared to controls. |
| 76. | Serum | ELISA | PAI-1 | 34 | 57 | x | x |  |  | Newly diagnosed | Not specified | Significantly higher levels in HGG patients as compared to LGG and controls. HGG patients with low levels (<10 ng/ml) had significantly longer OS vs. those with high levels (>10 ng/ml). |
| 77. | Serum | ELISA | YKL-40 | - | 343 | x | x |  |  | Mixed (183 newly diag-nosed) | Not specified | Increase in YKL-40 associated with increased risk of death for HGG patients. Levels lower in HGG patients with CR as compared to patients with radiographic disease (MR+SD+PD). Among patients with radiographic disease, YKL-40 levels were significantly different with tumor grade. HGG patients had significanlty higher levels compared to LGG patients. |
| 80. | Serum | ELISA | GFAP | 50 | 81 | x |  |  |  | Newly diagnosed | Prior to surgery | Significantly higher levels in patients with GBM as compared to patients with astrocytoma (WHO II and -III), AOD or MBL and controls. Levels correlate with tumor- and tumor necrosis volume in GBM patients. |
| 205. | Urine | Radio-immuno-assay | EGF | 62 | 38 | x |  |  | x | Not specified | Variable | Higher levels in patients with malignant glioma vs. meningioma patients and controls. Decreases upon radiation and surgery and increases upon progression. Levels might correlate with tumor volume. |
| 206. | Urine, Serum | ELISA | VEGF | - | 32 |  |  | x |  | Recurrent | Prior to treatment (metro-nomic chemo) | Levels significantly different between responding HGG patients (PR, MR and SD) and non-responders (PD). Levels predictive for response to a combination of thalidomide, celecoxib, etoposide and cyclophosphamide. |
| 82. | CSF | SELDI-TOF-MS, MALDI-TOF-MS | Carbonic anhydrase | 10 | 7 | x |  |  |  | Not specified | Not specified | Present in the CSF of GBM patients, absent in controls and in patients with WHO II glioma or reactive/  inflammatory CNS conditions. |
| 83. | CSF | 2DE-TOF/TOF-MS/MS | 130 proteins (incl. VEGF-B, PAI-1, Hapto-globin-1 and IL-17) | 10 | 32 | x |  |  |  | Mixed  (7 recur-rent) | Variable | 29 proteins differentially expressed between DA patients and controls,  66 proteins between patients with DA and AA, 132 between patients with DA and GBM and 89 between AA- and GBM patients. Used to build two prediction models, based on 13 and 31 proteins, respectively. |
|  | CSF | 2D-LC-MS/MS (cICAT) | 53 proteins (incl. VEGF, AHSG, NCAM) | 10 | 32 | x |  |  |  | Mixed  (7 recur-rent) | Variable | Able to discriminate patients with different glioma grades from each other, patients with MBL, patients with infectious CNS disease and controls. 30 proteins overlapped with  2DE-TOF/TOF-MS/MS data. |
|  | CSF | WB | VEGF-B, SPARC, FGF-14, Tau, β2-microglobulin, β-defensin-6 and -7, attractin | 10 | 32 | x |  |  |  | Mixed  (7 recur-rent) | Variable | Different levels among patients with different glioma grades and between glioma patients and controls. |
| 207. | Serum | Radial immunodiffusion | Immuno-suppres-sive acidic protein | 30 | 16 | x |  |  |  | Not specified | Prior to surgery | Signficantly higher levels in patients with glioma or MBL as compared to those with non-glial, benign tumors and healthy individuals. Also higher levels in patients with GBM vs. patients with other gliomas. |
| 208. | Serum | ELISA | IL-10,  IL-12 | 50 | 51 | x |  |  |  | Newly diagnosed | Not specified | Significantly higher IL-10 and lower  IL-12 levels in GBM patients as compared to patients with AA and controls. |
| 89. | CSF | 2DE, MALDI-TOF MS | α2-Hapto-globin, MHC-I, Ang-2b, Apolipo-protein  A-1 | 6 | 14 | x |  |  |  | Not specified | Prior to surgery | Upregulated levels in patients with GBM as compared to controls. |
|  | CSF | WB, ELISA | α2-Hapto-globin | 19, 26 | 120, 174 | x |  |  |  | Not specified | Prior to surgery | Higher levels in patients with GBM as compared to patients with DA or AA and controls |
| 90. | CSF | ELISA | MCP-1 | 7 | 27 | x |  |  |  | Not specified | Not specified | Higher levels in HGG patients as compared to patients with LGG and controls. |
| 94. | Plasma | ELISA | IGFBP-2 | 55 | 196 | x |  |  |  | Newly diagnosed | Prior to surgery | Higher levels in patients with HGG vs. LGG patients and controls. |
|  |  |  | IGFBP-2 | - | 52 |  | x |  |  | Newly diagnosed | Prior to surgery | Correlated with DFS. GBM patients with high levels (>650 ng/ml) had a significantly shorter DFS as compared to patients with low (<650 ng/ml) levels. |
|  |  |  | IGFBP-2 | - | 12-15 |  |  |  | x | Newly diagnosed | Change from baseline | Higher levels in GBM patients after recurrence as compared to pre-resection. |
| 95. | Plasma | ELISA | TIMP-1, MMP-9 | 59 | 285 | x |  |  |  | Newly diagnosed | Prior to surgery | Higher levels in glioma patients as compared to controls. Higher TIMP-1 levels in GBM vs. WHO II and III glioma. |
| 97. | CSF | HPLC-MS | 39 meta-bolites (incl. iso-citrate) | 7 | 10 | x |  |  |  | Mixed  (4 newly diag-nosed) | Not specified | Higher levels in HGG patients compared to controls. |
|  |  |  | 7 meta-bolites | 7 | 10 |  |  |  | x | Mixed  (4 newly diag-nosed) | Not specified | Differentially expressed beween nHGG and rHGG patients. Also, 6 other metabolites correlated with both T1 and FLAIR-MRI measurements. |
|  |  |  | 14 meta-bolites | 7 | 10 |  | x |  |  | Mixed  (4 newly diag-nosed) | Not specified | Correlated with the OS of patients with HGG. |
| 209. | Plasma | ELISA | TGFβ-1 (total; latent and active com-bined) | - | 25 |  |  |  | x | Not specified | Change from baseline | Levels 7 days after surgery correlated with tumor volume in HGG patients. Levels in both LGG- and HGG patients were reduced as compared to pre-resection values (baseline). |
| 98. | Urine | LC-MS/MS | 2HG | - | 26 | x |  |  |  | Not specified | Not specified | Significantly increased levels in urine of IDH1 wt patients as compared to patients with mutations in IDH1. The plasma/urine level provides even more significant results. |
| 102. | Serum | ELISA | GFAP,  S100b | 69 | 50 | x |  |  |  | Newly diagnosed | Prior to surgery | Higher levels of GFAP in patients with HGG as compared to controls Also, higher levels in GBM- vs. AA patients, but not elevated in MBL cases.  Higher levels of S100b in HGG- and MBL patients as compared to controls. Also, higher levels in GBM vs. AA patients, patients with meningioma and those with MBL. |
|  |  |  |  |  |  |  |  |  |  |  |  |  |
| 110. | Serum | Radial immuno-diffusion | Hapto-globin, α1-acid glyco-protein, α1-anti-trypsin | 9 | 16 | x |  |  |  | Not specified | Prior to treatment (surgery, chemo, RT) | Higher levels in GBM patients compared to controls. |
|  | Serum | Radial immunodiffusion | α1-acid glyco-protein | - | 16 |  | x |  |  | Not specified | Prior to treatment (surgery, chemo, RT) | GBM patients with an OS of <1 year have higher values as compared to patients surviving >1 year. |
| 210. | Serum | ELISA | CXCL-12 | 189 | 123 | x |  |  |  | Not specified | Not specified | Higher levels in patients with GBM or AA as compared to controls. |
| 115. | CSF | GC/MS | Citric-, isocitric-, fumaric acid | - | 22 | x |  |  |  | Not specified | Prior to surgery | Citric- and isocitric acid levels increased in patients with GBM vs. WHO II and III glioma. Also higher levels in IDH mutant patients as compared to IDH wt. Fumaric acid decreased in patients with GBM as compared to AA. |
|  |  |  | Citric-, isocitric acid | - | 22 |  |  |  | x | Not specified | Prior to surgery | In HGG patients, higher levels are observed in those with gadolinium-enhancing tumors. |
|  |  |  |  |  |  |  |  |  |  |  |  |  |
| 124. | CSF | ELISA | bFGF, VEGF | 10 | 26 | x | x |  | x | Newly diagnosed | During treatment (surgery) | Higher levels in HGG as compared to LGG and controls. Patients with above median levels (VEGF >15 ng/ml; bFGF > 41 ng/ml) have shorter OS. Also, a positive relation with tumor vascularity was observed. No such effects found for serum levels. |
| 211. | Serum | Turbi-dimetry | AHSG | 58 | 91 | x | x |  |  | Newly diagnosed | Prior to surgery | Serum levels significantly correlated with tumor grade. Serum AHSG <285 mg/l associated with shorter OS of GBM patients as compared to patients with lower levels. |
| 127. | Serum | ELISA | EGFR ECD | 50 | 65 | x | x |  |  | Newly diagnosed | After surgery | HGG patients with levels >80 ng/ml have worse OS as compared to patients with levels <80 ng/ml. |
| 128. | Serum | ELISA | VEGF, GM-CSF | 10 | 12 | x |  |  |  | Newly diagnosed | Not specified | Higher levels of VEGF and GM-CSF in patients with GBM vs. healthy controls. Also higher levels of VEGF in patients with MBL as compared to GBM. |
| 212. | Serum | ELISA | PBEF-1 | 9 | 80 | x |  |  |  | Not specified | Prior to surgery | Higher levels in GBM, AA and DA patients as compared to normal individuals. |
| 130. | Serum | ELISA | VEGF, sVEGFR-1, IL-6 | 60 | 40 | x |  |  |  | Newly diagnosed | Prior to surgery | Higher levels in patients with GBM as compared to controls. |
|  | Plasma | ELISA | TNFα | 60 | 40 | x |  |  |  | Newly diag-nosed | Prior to surgery | Higher levels in patients with GBM as compared to controls. |
| 141. | Plasma | ELISA | Thrombo-modulin | 19 | 22 | x |  |  |  | Not specified | Prior to surgery | Higher levels in patients with GBM as compared to controls. |
| 142. | CSF | ELISA | VEGF | 14 | 27 | x |  |  |  | Not specified | Not specified | Higher levels in HGG patients (25/27 GBM) compared to controls and patients with non-astrocytic CNS tumors. |
| 143. | CSF | NanoLC-MS/MS, WB | CypA, DDAH-1 | 4, 22 | 29, 27 | x |  |  |  | Not specified | Not specified | Detected in pediatric patients with DIPG, absent in most supratentorial glioma patients and controls. |
| 144. | CSF | Radio-immuno-assay | Ferritin | 20 | 16 | x |  |  |  | Not specified | Prior to surgery | Higher levels in patients with GBM compared to controls. |
| 145. | Plasma | Immuno-turbidi-metric assay | Fibro-nectin | 15 | 36 | x |  |  |  | Not specified | Not specified | Elevated in progressing, not stable, HGG patients and in LGG patients as compared to controls. |
| 147. | Plasma | ELISA | TGFb-1 and -2 (latent and active) | 26 | 21 | x |  |  |  | Not specified | Prior to surgery | Significantly elevated latent and active TGFb-1 and 2 in patients with GBM vs. healthy controls. Increased latent TGFb-2 in patients with GBM as compared to those with MS. |
|  |  |  | Latent TGFb-2 | - | 17 |  | x |  | x | Not specified | Change from baseline | Lower levels of latent TGFb-2 seven days post-resection as compared to pre-resection, but still higher levels compared to controls, and correlates with survival. Higher difference (>6 ng/ml) associated with longer OS of GBM patients. Also statistically significant correlation between higher pre-resection levels and longer OS. |
|  |  |  |  |  |  |  |  |  |  |  |  |  |
| 148. | CSF | MS/MS | AACT | - | 13 |  |  |  | x | Not specified | Not specified | Negative correlation with increasing tumor volume on MRI in patients with GBM. |
| 215. | Serum | Different techni-ques | Co-  enzyme Q,  uric acid, ferritin | 35 | 34 | x |  |  |  | Newly diagnosed | Prior to surgery | Higher (Coenzyme Q, ferritin) or lower (uric acid) levels in patients with GBM as compared to controls. |
| 151. | Plasma (MVs) | μNMR | EGFR, EGFRvIII,  PDPN | 12 | 24 | x |  |  |  | Mixed  (6 recur-rent) | Prior to surgery | Discriminates GBM patients from healthy controls. |
|  | Plasma (MVs) | μNMR | EGFR, EGFRvIII,  PDPN, IDH-R132H | - | 12 |  |  |  | x | Mixed | After CRT | Lower drug efficacy- and higher tumor progression index for non-responding compared to responding patients. |
| 153. | CSF | ELISA | MIC-1/  GDF-15 | - | 33 |  | x |  |  | Mixed | During treatment (surgery) | GBM (n=30 newly diagnosed, n=10 recurrent) patients with detectable levels of MIC-1/GDF-15 (>156 pg/ml) have shorter OS as compared to patients with undetectable levels. |
|  |  |  |  |  |  |  |  |  |  |  |  |  |
| 217. | Plasma | Multiplex sus-pension assay | TIMP-2, MIP-1β, RANTESGM-CSF | - | 28 |  |  | x |  | Recurrent | Prior to treatment (aflibercept) | Differential levels between GBM patients developing toxicity (TIMP-2, RANTES) or fatigue (MIP-1β, GM-CSF) and patients without these events during treatment. |
|  |  | Multiplex sus-pension assay | IL-1β, IL-6, IL-10,  IL-13 | - | 26 |  |  |  | x | Recurrent | Change from baseline | Differences in levels after 24h of treatment with aflibercept associated with the development of toxicity (IL-6, IL-10, IL-13) or fatigue (IL-1β, IL-6, IL-10) in patiens with GBM. |
|  |  | Multiplex sus-pension assay | MCP-3 | - | 21 |  |  |  | x | Recurrent | Change from baseline | Increased levels after 28 days of treatment with aflibercept associated with the development of toxicity in patiens with GBM. |
|  |  | ELISA | bFGF | - | 26 |  |  |  | x | Recurrent | Change from baseline | Differences in levels 24 hours after treatment with aflibercept associated with the development of toxicity in GBM patients. |
|  |  | ELISA | CA-9 | - | 28 |  |  | x |  | Recurrent | Prior to treatment (aflibercept) | Elevated in GBM patients experiencing toxicity vs. those without these events. |
|  |  | Lincoplex assay | E-selectin | - | 28 |  |  | x |  | Recurrent | Prior to treatment (aflibercept) | Elevated in GBM patients experiencing toxicity vs. those without these events. |
| 219. | Plasma | ELISA | Collagen IV | - | 28 |  |  |  | x | Recurrent | Change from baseline | Increases after 24 h of treatment associated with longer PFS of patients with GBM. |
| 157. | Serum | ELISA | TIMP-1 | 34 | 78 | x |  |  |  | Not specified | Prior to surgery | Higher levels in patients with GBM as compared to AA- and DA patients. |
|  |  |  | OPN | 22 | 126 | x |  |  |  | Not specified (≥30 newly diag-nosed) | Prior to surgery | Higher levels in patients with GBM as compared to AA, DA patients and normal individuals. |
|  |  |  | OPN | - | 30 |  | x |  |  | Newly diagnosed | Prior to surgery | GBM patients with higher OPN levels (>20 ng/ml) had shorter OS vs. those with lower OPN levels (<20 ng/ml). |
| 158. | Serum | Turbidi-metry | CRP | - | 165 |  | x |  |  | Not specified | Prior to surgery | Serum pre-treatment CRP levels correlated with survival time in HGG patients, but not for those with LGG. HGG patients with low CRP levels (<5 mg/l) had longer OS vs. patients with high CRP (>5 mg/l). |
| 165. | Plasma | ELISA | MMP-2 | - | 76 |  | x | x |  | Recurrent | Prior to treatment (beva-cizumab) | High MMP-2 level (>227.5 ng/ml) associated with response to bevacizumab in patients with glioma (majority HGG). High MMP-2 (>227.5 ng/ml) associated with longer OS and PFS as compared to patients with low (<227.5 ng/ml) levels. |
|  |  |  | MMP-9 | - | 26 |  | x | x |  | Recurrent | Prior to treatment (beva-cizumab) | Low MMP-9 levels (<235 ng/ml) associated with a higher probability of response to bevacizumab in HGG patients. Lower levels associated with increased OS and PFS. |
|  |  |  | VEGF | - | 26 |  |  |  | x | Recurrent | Change from baseline | A decrease in VEGF level after 15 days of treatment with bevacizumab is associated with increased OS and PFS of HGG patients. |
|  |  |  | uPA | - | 26 |  | x |  |  | Recurrent | Prior to treatment (beva-cizumab) | uPA levels are correlated with OS of patients with HGG. |
| 167. | Serum | ELISA | YKL-40 | 14 | 65 | x |  |  |  | Not specified | Not specified | Higher levels in patients with GBM as compared to WHO grade II or III glioma patients and controls. Also higher levels in patients with WHO II or III glioma patients compared to controls. |
| 171. | Serum | WB | NCAM (HMW and LMW) | 69 | 34 | x |  |  |  | Newly diagnosed | Prior to surgery | Glioma, meningioma, hypophysis adenoma and MBL patients have considerably more HMW species and few LMW species as compared to healthy controls. |
| 220. | Serum | ELISA | Galectin-1 | 43 | 25 | x |  |  | x | Newly diagnosed | Prior to surgery (during anesthesia) | Higher levels in nGBM patients as compared to healthy controls. Also higher at recurrence compared to control. |
| 175. | Serum | Immuno-lumino-metric assay | S100b | - | 20 |  | x |  |  | Mixed (≥10 recurrent) | Variable | Glioma patients with above median levels (>0.09 μg/l) had shorter OS. |
| 221. | Serum | ELISA | PGRN (pro-granulin) | 8 | 17 | x |  |  |  | Newly diagnosed | Not specified | Higher levels in patients with GBM as compared to healthy controls. |
| 188. | Plasma | Luminex | α2-micro-globulin, CA-19-9, Factor VII, SCF, MDC | 367 | 28 | x |  |  |  | Not specified | Prior to surgery | Higher (α2-micro-globulin, CA-19-9, Factor VII, SCF) or lower (MDC) levels in patients with HGG vs. controls. Results were confirmed in the serum of an independent patient group. Plasma and serum also contain several unique proteins discriminating HGG patients from controls. |
|  | Plasma | Luminex | GH, IL-16, VEGF | - | 28 |  | x |  |  | Not specified | Prior to surgery | Lower levels of VEGF (<507 pg/ml),  IL-16 (<219 pf/ml) and higher levels of GH (>50 ng/ml) associated with favourable OS for patients with HGG. |
| 198. | CSF | ELISA | OPN-FL, OPN-R, OPN-L, total OPN, VEGF, MIP-1β | 15 | 66 | x |  |  |  | Not specified | Not specified | Elevated levels in glioma- (OPN-R, OPN-L, total OPN, MIP-1β) or GBM patients only (OPN-FL, VEGF) as compared to controls. Levels of VEGF higher in patients with GBM vs., patients with WHO II or III gliomas. |
| 191 | CSF | ELISA | Tenascin | 57 | 19 | x |  |  | x | Not specified | Not specified | Elevated levels in HGA patients as compared to LGA. Also increased in LGA vs. non-astrocytic brain tumors. Negligible concentrations found in post-operative patients who underwent succesful surgery/RT/chemo. |
| 192. | Serum | ELISA | sIL-2Rα | 18 | 16 |  |  |  | x | Mixed | During disease course (not specified) | In 5 out of 16 HGG patients a significant increase in sIL-2Rα was detected before MRI could reveal actual tumor recurrence. |
| 194. | CSF | MALDI-TOF-MS | Cal-desmon (LMW) | 14 | 10 | x |  |  |  | Not specified | Not specified | Present in patients with glioma, absent from controls and MS patients. |
| 224. | Serum | ELISA | Cal-desmon (LMW) | 173 | 57 | x |  |  |  | Newly diagnosed | Prior to surgery | Higher levels in glioma patients as compared to controls (patients with non-glial tumors, other neurological diseases and healthy controls). |
| 223. | Plasma | Colori-metric nitrate/  nitrite assay | eNOS | 19 | 39 | x |  |  |  | Newly diagnosed | Prior to surgery | Higher in glioma patients compared to controls, patients with MBL and those with ischemic stroke. |
| 225. | Serum | ELISA | GASP-1 | 13 | 12 | x |  |  |  | Not specified | Not specified | Elevated levels in glioma patients as compared to controls. |
| 226. | Serum | Luminex | sCD14 | 737 | 1079 | x |  |  |  | Newly diagnosed | Not specified | sCD14 level higher in subjects with GBM or other gliomas as compared to controls. |
| 91. | Plasma | ELISA | GFAP  Neurogranin  BDNF  ICAM-5  MT3  SNCB  S100B  NSE | 15 | 34 | x |  |  |  | 23 GBM,  11 grade II | Prior to surgery | Of eight plasma CP markers, none of the proteins showed specificity for patients with glioblastoma. A significant difference between GBM patients and healthy controls of ICAM-5 was reported. No differences were found between grade IV and grade II glioma. |

**Supplemental table 2 - Comprehensive overview of circulating nucleic acids and circulating tumor cells in patients with diffuse glioma**

| **Ref.** | **Bio-**  **source** | **Assay** | **Type of origin**  **Marker** | **Population**  **size (n)** | | **Diagn** | **Progn** | **Pred** | **Monit** | **Patients** | **Samples** | **Main conclusions:** |
| --- | --- | --- | --- | --- | --- | --- | --- | --- | --- | --- | --- | --- |
|  |  |  |  | **Ctrl** | **Glioma** |  |  |  |  |  |  |  |
| 8. | Serum | Bisulfite  methy-lation PCR | ctDNA  MGMT, p16, DAPK, RASSF1A | - | 21 |  |  | x |  | Newly diagnosed | Prior to surgery | Serum *MGMT* methylation predictive for efficacy BCNU treatment, however not for temozolomide + cisplatin treatment. |
| 197. | Serum | Bisulfite  methy-lation PCR | ctDNA  MGMT | - | 37 |  | x | x |  | Newly diagnosed | Prior to surgery | Serum methylation of *MGMT* is an independent predictor of PFS and OS. High concordance between tissue and serum methylation status. |
| 18. | Whole blood | RT-PCR | CTC  GFAP | - | 20 |  |  |  |  | Astrocytoma (n=10) and GBM (n=10)  patients | During surgery | GFAP mRNA could not be detected in whole blood. |
| 19. | Plasma | digital droplet PCR | ctDNA  IDH1-R132H | 31 | 80 | x |  |  |  | Newly diagnosed | Prior to surgery | Detection of *IDH1 R132H* mutation in ctDNA (sens. 60%, spec. 100%). In LGG, increased tumor volume increases sensitivity. In HGG, increased contrast enhancement increases sensitivity. |
| 198. | Platelets | - | Counts | - | 153 |  | x |  |  | nGBM | Prior to surgery | Preoperative thrombocytosis is a prognostic factor associated with shorter survival time in patients with GBM. |
|  |  |  |  |  |  |  |  |  |  |  |  |  |
| 31. | Serum | Bisulfite  methy-lation PCR | ctDNA  ALU repeat sequence | 65 | 30 | x | x |  |  | Newly diagnosed | Pre- and intra-operative | Lower methylation level in patients with glioma compared to healthy controls. Able to discriminate between glioma grades. Good correlation between tissue and serum methylation status. Low methylation levels associated with decreased OS. |
| 48. | Plasma | Bisulfite methy-lation PCR | ctDNA MGMT | - | 58 |  | x | x | x | Newly diagnosed | Before CRT and during follow-up | High concordance between tissue and plasma *MGMT* promoter methylation. Trend for improved OS and PFS in methylated *MGMT* in plasma, less pronounced in HGG patients. Increase of cumulative incidence of *MGMT* promoter unmethylation during CRT treatment. |
| 74. | Plasma | qPCR | miRNA  miR-21 | 10 | 10 | x |  |  | x | Newly diagnosed | Prior to surgery | Increased levels of miR-21 in plasma from patients with GBM compared to healthy controls. Levels decrease during chemo-radiotherapy. Sharp increase of miR-21 ex-pression seen in patient who developed recurrence. No correlation with tumor volume and tissue levels. |
| 92. | Serum | Micro-satellite analysis & bisulfite methy-lation PCR | ctDNA  MGMT, PTEN,  LOH: 1p, 10p, 19q | - | 70 | x |  |  |  | Not specified | ~ 1 month after resection | Moderate sensitivity and high specificity of detecting a glioma when assessing clinical relevant tumor biomarkers in serum. |
| 96. | CSF, serum | Methy-lated DNA Immuno-precipi-tation | ctDNA  MGMT, p16/INK4a,  TIMP-3, THBS1 | 20 | 66 |  | x | x |  | Newly diagnosed | Photo-graphic diagnosis, pre-operative or before other treatment | Methylated serum markers are able to distinguish prognostic good and poor groups. High *MGMT* promoter methylation status associated with poorer outcome. |
| 103. | Serum | qPCR | miRNA  (miR-544) | - | 66 | x |  |  |  | Not specified | Not specified | miR-544 not useful in serum as a marker of progression from LGG to HGG. |
| 105. | Serum | Bisulfite methy-lation PCR | ctDNA  MGMT, RASSF1A, p15/INK4B,  p14/ARF | - | 17 glial, 16 non-glial | x |  |  |  | Newly diagnosed | Not specified | Moderate to high concor-dance between tumor tissue and serum methylation status. *RASSF1A* methylation significant different between glial and  non-glial tumors. |
| 106. | Serum EVs | TLDA, qRT-PCR | Exosomal small RNA  RNU6-1, miR 320,  miR 547-3p | 55 | 75 | x |  |  |  | Newly diagnosed | Pre-treatment | Serum exosomes from GBM patients contain elevated levels of RNU6-1. Levels of RNU6-1 combined with miR 320- and miR-547-3p have a moderate diagnostic value (AUC 0.7-0.8) to detect GBM. |
| 107. | Blood | Chroma-togenic and immuno-fluores-cence analysis | CTC  GFAP | - | 1 |  |  |  |  | Astrocytoma grade III with multiple bone metastases | During treatment of metastases | Despite multiple bone metastases, no CTCs were identified. |
| 116. | Throm-bocytes | RT-PCR, micro array | Thrombo-cyte RNA  EGFRvIII.  Several markers | 12 | 8 | x |  |  |  | Newly diagnosed | Prior to surgery | Detection of EGFRvIII mRNA in blood platelets from patients with GBM. Microarray biomarker profile distinguishes GBM patients from healthy controls. |
| 117. | Serum | Agilent micro arrays | Exosomal mRNA  B2M, TMSL3, RPS12, RPL11 *i.a.* | 7 | 9 | x |  |  |  | Newly diagnosed | Prior to surgery | Differential expressed genes (downregulated) in circulating exosomes of GBM patients. |
| 130. | Plasma | Flow Cytometry | Micro-particles | 40 | 22 |  |  |  |  | Newly diagnosed | During CRT | Increased levels of microparticles in patients with GBM compared to healthy controls. |
| 136. | Blood cells | micro array | miRNA  miR-128,  -342-3p,  -194, -628-3p, Life-6-3p, *i.a.* | 30 | 30 | x |  |  |  | Not specified | Not specified | Upregulation of miR-128, down-regulation of miR-342-3p. Diagnostic algorithm based on miRNA fingerprint. |
| 140. | Plasma | Long range PCR | ctDNA  EGFRvIII | - | 13 |  |  | x | x | Newly diagnosed | Prior to surgery + 3 weeks post-operative | Able to detect *EGFRvIII* ctDNA, levels associate with surgery. |
| 213. | Serum | ELISA | Microparticle activity | 20 | 61 |  |  |  | x | Newly diagnosed | Prior to surgery + follow-up until 7 months after surgery | Decrease in amount of active MPs after tumor resection, most notably after total resection. Tumor progression accompanied by increase of active MPs. |
| 214. | Serum | ELISA | Microparticle activity | 20 | 61 |  |  |  | x | Newly diagnosed | Prior to surgery + follow-up untill 7 months after surgery | After surgery increase of GFAP+ MPs, which do also increase during tumor progression. TF+ MPs increased in patients with VTE. No association with coagulant or fibrinolytic parameters. |
| 216 | CSF | qPCR | ctDNA  ALU115, ALU247 | 22 | 70 |  |  |  |  | Not specified | Not specified | Integrity of DNA in CSF superior compared to DNA in serum. |
| 152. | Plasma | qPCR | miR-454-3p | 100 | 100 | x | x |  | x | Newly diagnosed | Prior to and after surgery | Increased levels of miR-454-3p in glioma patients, even higher in HGG compared to LGG. High levels of miR-454-3p associated with worse prognosis. Levels drop following surgery. |
| 176. | Serum | Micro-satellite analysis & bisulfite methy-lation PCR | ctDNA  MGMT, p16/INK4a | 10 | 40 | x |  |  |  | Newly diagnosed | Not specified | Serum methylation of *p16* is not suitable for distinguishing astrocytoma from oligodendroglioma. *p16* methylation in serum of brainstem tumor patients may be useful to distinguishing glial from non-glial tumors. |
| 178. | Plasma | qPCR | miRNA  miR-15b,  -21, -128,  -181a/b/c,  -221, -222,  -342-3p | 10 | 50 | x |  |  | x | Not specified | Not specified, (GBM; prior to surgery and after CRT) | Ability to distinguish glioma grade. Normalization of biomarker levels after chemoradiation. |
| 222. | Plasma | Bisulfite methy-lation PCR | ctDNA  MGMT, p73, p16/INK4aRARb | - | 10 | x |  |  |  | Newly and recurrent diagnosed | Prior to surgery | Increased amount of circulating DNA in patients with glioma (no direct comparison to healthy controls) Promotor methylation detected in 60% of grade II-IV glioma |
| 190. | Serum | Solexa seq-uencing | miRNA  miR-15b,  -23a,  -133a,  -150, -197,  -497,  -548-5p | 137 | 177 | x |  |  | x | Newly diagnosed | Prior to surgery | Able to distinguish different intracranial lesions, miR-15b significantly associated with tumor grade. Elevation of markers after surgery, ability to detect an glioma associated miRNA fingerprint. |
| 104. | Blood cells | Telo-merase respon-sive adeno-viral probe | CTC | 30 | 11 | x |  |  | x | HGG | Prior to and after radiotherapy | CTCs are detectable in glioma patients. |
| 113. | Blood Cells | Density centrifu-gation, immune-cyto-chemistry | CTC | 28 of which 5 patients with brain meta-stesis | 141 | x |  |  |  | GBM | Prior surgery n=78, during n=32, after n=106 | CTCs are detactable in 20% of GBM patients and not in controls. No correlation with clinical outcome was found. |
| 162. | Whole blood | CTC-iChip | CTC | 6 | 33 | x |  |  |  | GBM | Prior to and after surgery | Isolation of CTCs is possible via size selecton followed by leukocyte depletion. CTCs identified in 13/33 patients, which contain a mesenchymal phenotype. More CTCs were isolated from patients with progressive disease compared to patients with stable disease. |
| 16. | Plasma | (Digital) PCR | ctDNA | - | 27 | x |  |  |  | Glioma |  | Only in minority (10%) of patients with a diffuse glioma, ctDNA could be measured. |

**Supplemental table 3 – Overview of sensitivity of circulating *MGMT* promoter methylation**

| Study [Reference] | Study population | Used method | Sensitivity of circulating *MGMT*  promotor methylation status |
| --- | --- | --- | --- |
| Balaña et al. 2003 [8] | GBM | Bisulfite methylation PCR | 5/6 = 83% |
| Weaver et al. 2006 [222] | OD, OA, GBM | Bisulfite methylation PCR | 2/4 = 50% |
| Wakabayashi et al. 2009 [176] | A, OD, OA, AA, AO, AO, GBM | Bisulfite methylation PCR | 14/18 = 78% |
| Balaña et al. 2011 [197] | GBM | Bisulfite methylation PCR | 9/15 = 60% |
| Lavon et al. 2010 [92] | AA | Bisulfite methylation PCR | 10/17 = 59% |
|  | OD |  | 7/15 = 47% |
| Lui et al. 2010 [96] | AA, AO, AOA, pGBM, sGBM | Methylated DNA immunoprecipitation | 47/64 = 73% |
| Majchrzak-Celinska et al.  2013 [105] | AA, GBM, rGBM | Bisulfite methylation PCR | 1/2 = 50%* |
| Fiano et al. 2014 [48] | A, OA, OD, AA, AO, AO, GBM | Bisulfite methylation PCR | 23/29 = 79% |
|  |  |  |  |

**Supplemental table 4 – Overview of discussed markers and their status as biomarkers (complementary to Figure 2)**

| **Circulating biomarker** | **Type of**  **Biomarker** | **Clinical Implications**  [Reference] |
| --- | --- | --- |
|  |  |  |
| Cathepsin D | Diagnostic | Yes (LGG vs. HGG) [200] |
|  | Prognostic | ND |
|  | Predictive | ND |
|  | Monitoring | ND |
|  |  |  |
| EGFRvIII Protein | Diagnostic | EGFRvIII: Yes [151, 202] |
|  | Prognostic | ND |
|  | Predictive | ND |
|  | Monitoring | ND |
|  |  |  |
| EGFRvIII Nucleic Acids | Diagnostic | Yes, ctDNA [140], EVs [155] and platelets [116] |
|  | Prognostic | ND |
|  | Predictive | ND |
|  | Monitoring | ND |
|  |  |  |
| G-CSF | Diagnostic | ND |
|  | Prognostic | ND |
|  | Predictive | Yes (prior to bevacizumab and irinotecan treatment) [46] |
|  | Monitoring | ND |
|  |  |  |
| GFAP | Diagnostic | Yes [24, 52, 68, 73, 80, 102] |
|  | Prognostic | ND |
|  | Predictive | ND |
|  | Monitoring | ND |
|  |  |  |
| IDH1 R132H NA | Diagnostic | Yes [19, 32] |
|  | Prognostic | ND |
|  | Predictive | ND |
|  | Monitoring | ND |
|  |  |  |
| IL-2 | Diagnostic | Yes (healthy controls vs. LGG vs. HGG) [59] |
|  | Prognostic | ND |
|  | Predictive | ND |
|  | Monitoring | ND |
|  |  |  |
| IL-8 | Diagnostic | Yes (healthy control vs. LGG vs. HGG, also HGG vs. metastastic brain lesion) [28, 73, 203] |
|  | Prognostic | ND |
|  | Predictive | Yes, prior to bevacizumab + irinotecan treatment [46] |
|  | Monitoring | Yes, during cediranib treatment [11, 12] |
|  |  |  |
| Methylated ALU Repeats | Diagnostic | Yes [31] |
|  | Prognostic | Yes [31] |
|  | Predictive | ND |
|  | Monitoring | ND |
|  |  |  |
| MGMT Promoter Methylation | Diagnostic | Yes [96, 176] |
|  | Prognostic | Yes [48, 96, 197] |
|  | Predictive | Yes, for BCNU treatment [8] |
|  | Monitoring | Yes, CRT treatment [48] |
|  |  |  |
| miR-21 | Diagnostic | Yes [74, 178] |
|  | Prognostic | ND |
|  | Predictive | ND |
|  | Monitoring | Yes [74, 178] |
|  |  |  |
| miR-454-3p | Diagnostic | Yes (healthy control vs. LGG vs. HGG) [152] |
|  | Prognostic | Yes [152] |
|  | Predictive | ND |
|  | Monitoring | Yes (following surgery) [152] |
|  |  |  |
| MMP-2 | Diagnostic | ND |
|  | Prognostic | Yes [165] |
|  | Predictive | Yes, bevacizumab + irinotecan treatment [165] |
|  | Monitoring | Yes, cediranib treatment [11] and chemoRT + vatalanib [56] |
|  |  |  |
| MMP-9 | Diagnostic | Yes (healthy controls vs. LGG and GBM) [95] |
|  | Prognostic | ND |
|  | Predictive | Yes, bevacizumab + irinotecan treatment [165] |
|  | Monitoring | Yes, aflibercept treatment [62], CRT + vatalanib treatment [56], monitoring complete response [67] |
|  |  |  |
| OPN | Diagnostic | Yes (healthy controls and WHO grade III glioma vs. GBM) [157, 189] |
|  | Prognostic | Yes [63, 157] |
|  | Predictive | ND |
|  | Monitoring | Yes [63] |
|  |  |  |
| PAI-1 | Diagnostic | Yes (healthy controls vs. LGG vs. HGG) [76] |
|  | Prognostic | Yes [76] |
|  | Predictive | ND |
|  | Monitoring | ND |
|  |  |  |
| PlGF | Diagnostic | Yes (HGG vs. metastatic brain tumor) [73] |
|  | Prognostic | ND |
|  | Predictive | Yes, aflibercept treatment [62] |
|  | Monitoring | Yes, cediranib treatment [11, 12, 13] |
|  |  |  |
| small RNAs sets | Diagnostic | Yes, exosomal RNU6-1, miR-320 and miR-547-3p [106],  miRNA biomarker sets although conflicting results [136, 178, 190] |
|  | Prognostic | ND |
|  | Predictive | ND |
|  | Monitoring | Yes, biomarker set (miR-23, -150, -197 and -548b-5p) [190] |
|  |  |  |
| TGF-b 1 and 2 | Diagnostic | Yes [127, 147, 202] |
|  | Prognostic | Yes [147] |
|  | Predictive | ND |
|  | Monitoring | Yes (following surgery) [147, 209] |
|  |  |  |
| TIMP1 | Diagnostic | Yes (healthy controls vs. WHO grade III glioma vs. GBM) [39, 95, 157] |
|  | Prognostic | Yes [39] |
|  | Predictive | ND |
|  | Monitoring | ND |
|  |  |  |
| TNF-a | Diagnostic | Yes (healthy controls vs. LGG vs. HGG) [2, 28, 59, 130] |
|  | Prognostic | ND |
|  | Predictive | ND |
|  | Monitoring | ND |
|  |  |  |
| VEGF | Diagnostic | Conflicting evidence [2, 28, 37, 39, 71, 83, 128, 130, 189] |
|  | Prognostic | Yes [188] |
|  | Predictive | ND |
|  | Monitoring | Conflicting evidence, bevacizumab + irinotecan treatment in rHGG [46, 139, 165] |
|  |  |  |
| sVEGFR-1 | Diagnostic | Yes (healthy controls vs. GBM) [130] |
|  | Prognostic | ND |
|  | Predictive | ND |
|  | Monitoring | Yes, cediranib treatment [12] and CRT and vatalanib treatment [56] |
|  |  |  |
| YKL-40 | Diagnostic | Yes (healthy controls vs. LGG vs. HGG) [15, 77, 167] |
|  | Prognostic | Yes [15, 67, 77] |
|  | Predictive | ND |
|  | Monitoring | Yes [15, 67, 77] |

**References**

196. Alper O, Stetler-Stevenson WG, Harris LN, Leitner WW, Ozdemirli M, Hartmann D, Raffeld M, Abu-Asab M, Byers S, Zhuang Z, Oldfield EH, Tong Y, Bergmann-Leitner E, Criss WE, Nagasaki K, Mok SC, Cramer DW, Karaveli FS, Goldbach-Mansky R et al. (2009) Novel anti-filamin-A antibody detects a secreted variant of filamin-A in plasma from patients with breast carcinoma and high-grade astrocytoma. Cancer Sci 100:1748–56. doi: 10.1111/j.1349-7006.2009.01244.x

197. Balańa C, Carrato C, Ramírez JL, Cardona AF, Berdiel M, Sánchez JJ, Tarón M, Hostalot C, Musulen E, Ariza A, Rosell R (2011) Tumour and serum MGMT promoter methylation and protein expression in glioblastoma patients. Clin Transl Oncol 13:677–85. doi: 10.1007/s12094-011-0714-x

198. Brockmann M a, Giese A, Mueller K, Kaba FJ, Lohr F, Weiss C, Gottschalk S, Nolte I, Leppert J, Tuettenberg J, Groden C (2007) Preoperative thrombocytosis predicts poor survival in patients with glioblastoma. Neuro Oncol 9:335–42. doi: 10.1215/15228517-2007-013

199. Doroudchi M, Pishe ZG, Malekzadeh M, Golmoghaddam H, Taghipour M, Ghaderi A (2013) Elevated Serum IL-17A but not IL-6 in Glioma Versus Meningioma and Schwannoma. Asian Pacific J Cancer Prev 14:5225–5230. doi: 10.7314/APJCP.2013.14.9.5225

200. Fukuda ME, Iwadate Y, Machida T, Hiwasa T, Nimura Y (2005) Cathepsin D Is a Potential Serum Marker for Poor Prognosis in Glioma Patients Cathepsin D Is a Potential Serum Marker for Poor Prognosis in Glioma Patients. 5190–5194.

201. Gollapalli K, Ray S, Srivastava R, Renu D, Singh P, Dhali S, Bajpai Dikshit J, Srikanth R, Moiyadi A, Srivastava S (2012) Investigation of serum proteome alterations in human glioblastoma multiforme. Proteomics 12:2378–90. doi: 10.1002/pmic.201200002

202. Graner MW, Alzate O, Dechkovskaia AM, Keene JD, Sampson JH, Mitchell D a, Bigner DD (2009) Proteomic and immunologic analyses of brain tumor exosomes. FASEB J 23:1541–57. doi: 10.1096/fj.08-122184

203. Hands JR, Abel P, Ashton K, Dawson T, Davis C, Lea RW, McIntosh AJS, Baker MJ (2013) Investigating the rapid diagnosis of gliomas from serum samples using infrared spectroscopy and cytokine and angiogenesis factors. Anal Bioanal Chem 405:7347–55. doi: 10.1007/s00216-013-7163-z

204. Iłzecka J, Iłzecki M (2006) APRIL is increased in serum of patients with brain glioblastoma multiforme. Eur Cytokine Netw 17:276–80.

205. Kanno H, Chiba Y, Kyuma Y, Hayashi a, Abe H, Takada H, Kim I, Yamamoto I (1993) Urinary epidermal growth factor in patients with gliomas: significance of the factor as a glial tumor marker. J Neurosurg 79:408–13. doi: 10.3171/jns.1993.79.3.0408

206. Kesari S, Schiff D, Doherty L, Gigas DC, Batchelor TT, Muzikansky A, O’Neill A, Drappatz J, Chen-Plotkin AS, Ramakrishna N, Weiss SE, Levy B, Bradshaw J, Kracher J, Laforme A, Black PM, Folkman J, Kieran M, Wen PY (2007) Phase II study of metronomic chemotherapy for recurrent malignant gliomas in adults. Neuro Oncol 9:354–63. doi: 10.1215/15228517-2007-006

207. Kikuchi K, Gotoh H, Kowada M (1987) Immunosuppressive acidic protein in patients with brain tumours: A preliminary report. Acta Neurochir (Wien) 86:42–49. doi: 10.1007/BF01419503

208. Kumar R, Kamdar D, Madden L, Hills C, Crooks D, O’Brien D, Greenman J (2006) Th1/Th2 cytokine imbalance in meningioma, anaplastic astrocytoma and glioblastoma multiforme patients. Oncol Rep 15:1513–6.

209. Loh J-K, Lieu A-S, Su Y-F, Cheng C-Y, Tsai T-H, Lin C-L, Lee K-S, Hwang S-L, Kwan A-L, Wang C-J, Hong Y-R, Chio C-C, Howng S-L (2013) Plasma levels of transforming growth factor-beta 1 before and after removal of low- and high-grade astrocytomas. Cytokine 61:413–8. doi: 10.1016/j.cyto.2012.11.011

210. Moosavi SR, Khorramdelazad H, Amin M, Fatahpoor S, Moogooei M, Karimabad MN, Paghale MJ, Vakilian A, Hassanshahi G (2013) The SDF-1 3’A genetic variation is correlated with elevated intra-tumor tissue and circulating concentration of CXCL12 in glial tumors: a study on Iranian anaplastic astrocytoma and glioblastoma multiforme patients. J Mol Neurosci 50:298–304. doi: 10.1007/s12031-013-9954-2

211. Petrik V, Saadoun S, Loosemore A, Hobbs J, Opstad KS, Sheldon J, Tarelli E, Howe F a, Bell BA, Papadopoulos MC (2008) Serum alpha 2-HS glycoprotein predicts survival in patients with glioblastoma. Clin Chem 54:713–22. doi: 10.1373/clinchem.2007.096792

212. Reddy PS, Umesh S, Thota B, Tandon A, Pandey P, Hegde AS, Balasubramaniam A, Chandramouli BA, Santosh V, Rao MRS, Kondaiah P, Somasundaram K (2008) PBEF1/NAmPRTase/Visfatin: a potential malignant astrocytoma/glioblastoma serum marker with prognostic value. Cancer Biol Ther 7:663–8.

213. Sartori MT, Della Puppa A, Ballin A, Saggiorato G, Bernardi D, Padoan A, Scienza R, d’Avella D, Cella G (2011) Prothrombotic state in glioblastoma multiforme: an evaluation of the procoagulant activity of circulating microparticles. J Neurooncol 104:225–31. doi: 10.1007/s11060-010-0462-8

214. Sartori MT, Della Puppa A, Ballin A, Campello E, Radu CM, Saggiorato G, d’Avella D, Scienza R, Cella G, Simioni P (2013) Circulating microparticles of glial origin and tissue factor bearing in high-grade glioma: a potential prothrombotic role. Thromb Haemost 110:378–85. doi: 10.1160/TH12-12-0957

215. Schwartzbaum JA, Cornwell DG (2000) Oxidant stress and glioblastoma multiforme risk: serum antioxidants, gamma-glutamyl transpeptidase, and ferritin. Nutr Cancer 38:40–9. doi: 10.1207/S15327914NC381_7

216. Shi W, Lv C, Qi J, Zhao W, Wu X, Jing R, Wu X, Ju S, Chen J (2012) Prognostic value of free DNA quantification in serum and cerebrospinal fluid in glioma patients. J Mol Neurosci 46:470–5. doi: 10.1007/s12031-011-9617-0

217. Shonka N, Piao Y, Gilbert M, Yung A, Chang S, DeAngelis LM, Lassman AB, Liu J, Cloughesy T, Robins HI, Lloyd R, Chen A, Prados M, Wen PY, Heymach J, de Groot J (2013) Cytokines associated with toxicity in the treatment of recurrent glioblastoma with aflibercept. Target Oncol 8:117–25. doi: 10.1007/s11523-013-0254-0

218. Da Silveira FDCA, Lopes BDA, da Fonseca CO, Quirico-Santos T, de Palmer Paixão ICN, de Amorim LMDF (2012) Analysis of EGF+61A>G polymorphism and EGF serum levels in Brazilian glioma patients treated with perillyl alcohol-based therapy. J Cancer Res Clin Oncol 138:1347–54. doi: 10.1007/s00432-012-1203-5

219. Sorensen a G, Batchelor TT, Zhang W-T, Chen P-J, Yeo P, Wang M, Jennings D, Wen PY, Lahdenranta J, Ancukiewicz M, di Tomaso E, Duda DG, Jain RK (2009) A “vascular normalization index” as potential mechanistic biomarker to predict survival after a single dose of cediranib in recurrent glioblastoma patients. Cancer Res 69:5296–300. doi: 10.1158/0008-5472.CAN-09-0814

220. Verschuere T, Van Woensel M, Fieuws S, Lefranc F, Mathieu V, Kiss R, Van Gool SW, De Vleeschouwer S (2013) Altered galectin-1 serum levels in patients diagnosed with high-grade glioma. J Neurooncol 115:9–17. doi: 10.1007/s11060-013-1201-8

221. Wang M, Li G, Yin J, Lin T, Zhang J (2012) Progranulin overexpression predicts overall survival in patients with glioblastoma. Med Oncol 29:2423–31. doi: 10.1007/s12032-011-0131-6

222. Weaver KD, Grossman S a, Herman JG (2006) Methylated tumor-specific DNA as a plasma biomarker in patients with glioma. Cancer Invest 24:35–40. doi: 10.1080/07357900500449546

223. Zheng P-P, Hop WC, Luider TM, Sillevis Smitt P a E, Kros JM (2007) Increased levels of circulating endothelial progenitor cells and circulating endothelial nitric oxide synthase in patients with gliomas. Ann Neurol 62:40–8. doi: 10.1002/ana.21151

224. Zheng P-P, Hop WC, Sillevis Smitt PAE, van den Bent MJ, Avezaat CJJ, Luider TM, Kros JM (2005) Low-molecular weight caldesmon as a potential serum marker for glioma. Clin Cancer Res 11:4388–92. doi: 10.1158/1078-0432.CCR-04-2512

225. Zheng X, Chang F, Zhang X, Rothman VL, Tuszynski GP (2012) G-protein coupled receptor-associated sorting protein 1 (GASP-1), a ubiquitous tumor marker. Exp Mol Pathol 93:111–5. doi: 10.1016/j.yexmp.2012.03.013

226. Zhou M, Wiemels JL, Bracci PM, Wrensch MR, McCoy LS, Rice T, Sison JD, Patoka JS, Wiencke JK (2010) Circulating levels of the innate and humoral immune regulators CD14 and CD23 are associated with adult glioma. Cancer Res 70:7534–42. doi: 10.1158/0008-5472.CAN-10-0815
